# A risk score combining co-expression modules related to myeloid cells and alternative splicing associates with response to PD-1/PD-L1 blockade in non-small cell lung cancer

**DOI:** 10.3389/fimmu.2023.1178193

**Published:** 2023-07-10

**Authors:** Yichao Han, Si-Yang Maggie Liu, Runsen Jin, Wangyang Meng, Yi-Long Wu, Hecheng Li

**Affiliations:** ^1^ Department of Thoracic Surgery, Ruijin Hospital, Shanghai Jiao Tong University School of Medicine, Shanghai, China; ^2^ Department of Hematology, the First Affiliated Hospital, Jinan University, Guangzhou, China; ^3^ Guangdong Lung Cancer Institute, Guangdong Provincial People’s Hospital (Guangdong Academy of Medical Sciences), Southern Medical University, Guangzhou, China

**Keywords:** non-small cell lung cancer, immune checkpoint blockade, responsiveness, transcriptomic analysis, myeloid cells, alternative splicing

## Abstract

**Background:**

Comprehensive analysis of transcriptomic profiles of non-small cell lung cancer (NSCLC) may provide novel evidence for biomarkers associated with response to PD-1/PD-L1 immune checkpoint blockade (ICB).

**Methods:**

We utilized weighted gene co-expression network analysis (WGCNA) to analyze transcriptomic data from two NSCLC datasets from Gene Expression Omnibus (GSE135222 and GSE126044) that involved patients received ICB treatment. We evaluated the correlation of co-expression modules with ICB responsiveness and functionally annotated ICB-related modules using pathway enrichment analysis, single-cell RNA sequencing, flow cytometry and alternative splicing analysis. We built a risk score using Lasso-COX regression based on hub genes from ICB-related modules. We investigated the alteration of tumor microenvironment between high- and low- risk groups and the association of the risk score with previously established predictive biomarkers.

**Results:**

Our results identified a black with positive correlation and a blue module with negative correlation to ICB responsiveness. The black module was enriched in pathway of T cell activation and antigen processing and presentation, and the genes assigned to it were consistently expressed on myeloid cells. We observed decreased alternative splicing events in samples with high signature scores of the blue module. The Lasso-COX analysis screened out three genes (*EVI2B*, *DHX9*, *HNRNPM*) and constructed a risk score from the hub genes of the two modules. We validated the predictive value of the risk score for poor response to ICB therapy in an in-house NSCLC cohort and a pan-cancer cohort from the KM-plotter database. The low-risk group had more immune-infiltrated microenvironment, with higher frequencies of precursor exhausted CD8^+^ T cells, tissue-resident CD8^+^ T cells, plasmacytoid dendritic cells and type 1 conventional dendritic cells, and a lower frequency of terminal exhausted CD8^+^ T cells, which may explain its superior response to ICB therapy. The significant correlation of the risk score to gene signature of tertiary lymphoid structure also implicated the possible mechanism of this predictive biomarker.

**Conclusions:**

Our study identified two co-expression modules related to ICB responsiveness in NSCLC and developed a risk score accordingly, which could potentially serve as a predictive biomarker for ICB response.

## 1 Introduction

Lung cancer is the leading cause of cancer-related deaths globally, with an estimated 2.2 million new cases and 1.8 million deaths in 2020 (1). Non-small cell lung cancer (NSCLC) is the major subtype of lung cancer, accounting for approximately 80% of all cases (2). In recent years, there have been significant breakthroughs in the use of immune checkpoint blockade (ICB) as a form of immunotherapy for NSCLC. Therapeutic antibodies against programmed cell death-1 (PD-1) and programmed cell death-ligand 1 (PD-L1) are one of the most important forms of ICB and are approved as the first-line treatment for patients with advanced or metastatic NSCLC (3). PD-(L)1 blockade has shown remarkable survival benefits in NSCLC (4). However, durable responses to anti-PD-(L)1 therapy are limited to a subset of patients, leading to ongoing trials evaluating ICB-based combination strategies (5). Therefore, it remains a pressing challenge to characterize the underlying factors associated with ICB response and identify the optimal biomarkers to screen responsive patients, which would improve treatment outcomes and reduce unnecessary side effects.

PD-L1 expression is a well-established biomarker for predicting response to PD-(L)1 blockade and is extensively used in clinical practice (6). According to multiple guidelines, patients with PD-L1 expression >= 50% but lacking driver mutations should receive anti-PD-(L)1 treatment (3, 7). However, PD-L1 expression is an imperfect predictor of ICB response. For instance, some PD-L1-negative patients may benefit from ICB therapy, while some PD-L1-positive patients may not have a durable response (8). Another well-known biomarker is tumor mutational burden (TMB), which is a measure of the number of somatic mutations per megabase (Mb) in a cancer (9). TMB-high tumors are thought to produce more neoantigens that are recognized by the immune system and have a better response to ICB therapy (10). However, the evidence supporting TMB as a predictor of ICB efficacy in NSCLC is mostly retrospective and TMB has not yet been standardized or widely adopted in clinical practice (11). Some studies have investigated biomarkers at the transcriptomic level, such as a T-cell-inflamed gene-expression profile (GEP), which was calculated by summing the normalized expression of 18 genes associated with T cell activation and inflammation (12). Several pan-cancer studies, including one involving NSCLC patients, have validated the predictive value of T-cell-inflamed GEP (13, 14). Other transcriptomic signatures, such as effector T cell signature and IFNγ signature, have also been investigated as potential biomarkers for ICB response (15, 16). Nevertheless, these signatures are based on screening results of hundreds of transcripts or pre-identified gene sets, and may not capture the full range of transcriptomic features related to ICB responsiveness.

Herein, our aim was to identify biomarkers that could predict the response to PD-(L)1 blockade in NSCLC by analyzing RNA sequencing (RNA-seq) data in an unsupervised manner. We integrated two RNA-seq datasets of NSCLC from Gene Expression Omnibus (GEO), specifically GSE135222 and GSE126044, where samples were collected before anti-PD-(L)1 treatment and clinical data regarding response to ICBs were available (17, 18). Using weighted gene co-expression network analysis (WGCNA), we identified two ICB-relevant modules. One module was related to myeloid cells and had a positive correlation to ICB responsiveness, while the other module was related to alternative splicing and had a negative correlation. To further characterize the biological details of the ICB-relevant modules, we conducted pathway enrichment analysis and alternative splicing analysis using TCGA-NSCLC datasets (TCGA-LUAD and TCGA-LUSC). Additionally, we analyzed the cellular expression distribution by utilizing data of single-cell RNA (scRNA) sequencing from GSE148071 (19) and flow cytometry that we conducted ourselves. Subsequently, we used Lasso-COX regression to screen out three hub genes (*EVI2B*, *DHX9*, *HNRNPM*) from the two modules and created a risk score that could separate patients with NSCLC into high-risk and low-risk groups for poor ICB response. The risk score was validated using our ICB-treated NSCLC cohort in Guangdong Lung Cancer Institute (GLCI) (20) and a pan-cancer immunotherapy cohort sourced from the KM-plotter database (21). We analyzed the tumor microenvironment (TME) and found that low-risk samples had more immune cell infiltration, with increased frequencies of precursor exhausted CD8^+^ T cells (Tpex), tissue-resident CD8^+^ T cells (Trm) and decreased frequency of terminal exhausted CD8^+^ T cells (Tex), contributing to the mechanism of ICB responsiveness. Furthermore, we found a close association between the risk score and the signature of tertiary lymphoid structure (TLS) through correlation analysis. Our study sheds light on the co-expression modules associated with ICB responsiveness and proposed a novel risk score, which could help us understand the mechanism of ICB treatment and optimize the biomarkers to predict response to ICB therapy in NSCLC.

## 2 Materials and methods

### 2.1 Data collection

The RNA-seq data of two training datasets, GSE135222 (n=27) and GSE126044 (n=16), were obtained from GEO. Pre-treatment NSCLC samples were analyzed in these studies, and clinical data on ICB response and progression-free survival (PFS) were available (**Supplementary Table 1**). Validation datasets were used to perform functional annotation of key modules. We downloaded RNA-seq data, somatic variant data and clinical information of TCGA-LUAD (n=513) and TCGA-LUSC (n=501) from GDC TCGA data portal (https://portal.gdc.cancer.gov/) and UCSC Xena browser (https://xena.ucsc.edu/) (**Supplementary Table 2**). We downloaded alternative splicing data from TCGA SpliceSeq (https://bioinformatics.mdanderson.org/TCGASpliceSeq/) (22). We also obtained RNA-seq data of Genotype-Tissue Expression (GTEx) project from UCSC Xena browser. For scRNA-seq data, we used GSE148071 (n=42) from GEO, which included advanced NSCLC samples. We validated the correlation with ICB responsiveness in two transcriptomic datasets. We collected one dataset of pre-treatment RNA-seq data and corresponding clinical information from 56 NSCLC patients with anti-PD-(L)1 monotherapy from GLCI, as described previously (**Supplementary Table 3**) (20). The other dataset was a pan-caner cohort from Kaplan-Meier Plotter database (KM-plotter, https://kmplot.com/analysis/), which contained 8 tumor types (melanoma, head and neck squamous cell carcinoma, NSCLC, bladder cancer, glioblastoma, hepatocellular carcinoma, esophageal adenocarcinoma and urothelial cancer) (21). We filtered samples in KM-plotter by options of pre-treatment and anti-PD-(L)1 therapy.

### 2.2 Definition of anti-PD-(L)1 responsiveness

ICB response data in GSE135222 and GSE126044 were available in the form of response by RECIST 1.1 (23) and PFS. We defined responsiveness as the best overall response of complete response (CR), partial response (PR) or stable disease (SD) >= 6 months, while irresponsiveness as progressive disease (PD) or stable disease (SD) < 6 months. ICB response data in the form of overall survival (OS) was available in the GLCI NSCLC and KM-plotter pan-cancer validation datasets.

### 2.3 RNA-seq normalization and integration

All RNA-seq data were normalized to TPM (transcript per million). To integrate the two training datasets, we used the ‘ComBat’ function in the sva R package (version 3.46.0) to correct for batch effects (24). We performed principal component analysis (PCA) to evaluate the successful batch correction. For WGCNA analysis, we used genes with the top 75% expression variance across samples, which resulted in 3316 genes being kept as input.

### 2.4 Co-expression network construction

WGCNA was analyzed using the R package WGCNA (version 1.71) (25). To perform quality control of samples and genes, we used the WGCNA function ‘goodSamplesGenes’. We checked sample outliers using hierarchical clustering via the R package flashClust (version 1.1.2) (26). We applied the WGCNA function ‘pickSoftThreshold’ to pick the optimal soft thresholding power (β) ranging from 1 to 30 to achieve scale free topology (R^2^ > 0.85) with networkType set as “signed”. A weighted adjacency matrix was calculated using the WGCNA function ‘adjacency’ with parameters set as follows: power=β, networkType=“signed”, corType=“bicor”. We used biweight midcorrelation (bicor) because it is considered more robust in analyzing similarity in gene expression matrices with less sensitivity to outliers (26). The adjacency matrix was converted to a topological overlap matrix (TOM) based dissimilarity using the function ‘TOMdist’. We conducted hierarchical clustering for dissimilarity followed by dynamic tree cutting using the ‘cutreeDynamic’ function to identify modules with similar expression profiles. Module eigengene (ME) was computed using the WGCNA function ‘moduleEigengenes’ as the first principal component of the expression matrix of each module. Similar modules were combined using the WGCNA function ‘mergeCloseModules’ with a cutHeight value of 0.25. We performed module preservation using the WGCNA function ‘modulePreservation’.

### 2.5 Identification of modules related to ICB responsiveness and their hub genes

To quantify the correlation of modules to clinical traits, we calculated the Pearson correlation between module eigengenes and each clinical trait. We further analyzed modules with significant relevance to anti-PD-(L)1 responsiveness. Specifically, we calculated module membership or eigengene-based connectivity (kME) as the correlation of each gene to the corresponding module eigengene, and gene significance (GS) as the Pearson correlation between genes and ICB responsiveness. The kME and GS of module genes were displayed by the WGCNA function ‘verboseScatterplot’. Genes with kME > 0.5 and |GS| > 0.35 were defined as hub genes, which are most interconnected in a module and related to ICB responsiveness. Top 30 hub genes were visualized using the software Cytoscape (version 3.8.2) (27). We performed tumor purity adjustment by calculating the partial Pearson correlation using the R package ppcor (version 1.1) (28).

### 2.6 Gene set variation analysis (GSVA) and survival analysis

GSVA was used to evaluate the module score of samples via the R package GSVA (version 1.42.0) (29). Kaplan-Meier analysis was performed based on the module eigengenes or GSVA scores using the R package survminer (version 0.4.9).

### 2.7 Pathway enrichment analysis and gene set enrichment analysis

We analyzed Gene Ontology (GO) and Kyoto Encyclopedia of Genes and Genomes (KEGG) using the ‘enrichGO’ and ‘enrichKEGG’ functions from the R package clusterProfiler (version 4.2.2) based on the genes assigned to the ICB-related modules (30). We performed GSEA analysis against GO and KEGG using the clusterProfiler function ‘gseGO’ and ‘gseKEGG’ with module genes ranked by their kME values.

### 2.8 scRNA-seq analysis

For scRNA-seq analysis, we used the R package Seurat (version 4.1.0) (31) according to the original paper (19). Firstly, we normalized the library size using the Seurat function ‘NormalizeData’. We then detected the top 2000 most variable genes using the function ‘FindVariableFeatures’, and scaled the variable genes by regressing out the unwanted sources of variation via the function ‘ScaleData’. We performed PCA analysis using the function ‘RunPCA’ and selected the top PCs to calculate nearest neighbors and cluster cells using the Seurat function ‘FindNeighbors’ and ‘FindClusters’. Cell clusters were visualized with the uniform manifold approximation and projection (UMAP) map and annotated by marker genes suggested in the original paper. Gene expression of each cluster was demonstrated by dot plots and heatmaps using the Seurat function ‘DotPlot’ and ‘DoHeatmap’. Analysis of subpopulations was done in similar steps. If necessary, we corrected batch effect using the R package harmony (version 0.1.1) (32). Similar cells were grouped as metacells based on the MetaCell algorithm using the ‘MetacellsByGroups’ function from the R package hdWGCNA (version 0.2.11) (33), therefore solving the problem of transcript drop-out. We performed single-cell level correlation analysis based on the metacell gene matrix.

### 2.9 Human specimen and ethics statement

The collection of specimen complies with all related ethical regulations and was approved by the Ethics Committee of Ruijin Hospital, Shanghai Jiao Tong University School of Medicine (KY2018-104). We obtained tumor tissues that were surgically removed from 10 patients who had been pathologically diagnosed with NSCLC. These patients did not receive any treatment before surgery (**Supplementary Table 4**).

### 2.10 Tissue processing and flow cytometry

Fresh tumor tissues were processed immediately after collection. The tissues were mechanically minced and subjected to enzymatic digestion using 1 mg/mL type IV collagenase (Worthington Biochemical, NJ, USA) with 150 μg/mL DNase I (Worthington Biochemical, NJ, USA) for 60 min in a 37°C rotating incubator. The dissociated samples were filtered using a 70 μm strainer, washed and resuspended with fluorescence activated cell sorting (FACS) buffer. Cells were incubated with 5 μL TruStain FcX block antibody (BioLegend, CA, USA) in 100 µL FACS buffer for 10 min on ice and then stained with fluorochrome-conjugated antibodies and Fixable Viability Dye eFluor 780 (Invitrogen, CA, USA) for 30 min on ice. Antibodies used were: anti-human CD45–FITC(HI30), CD3–BV650(OKT3), CD19–PE/Dazzle(HIB19), CD15–AF700(HI98), CD14–BV605(M5E2), HLA-DR–BV785(L243), CD11c–BV510(3.9), CD53–PE(HI29), all from BioLegend, and Paired immunoglobulin-like receptor α (PILRα)–AF647(2175D) from R&D (MN, USA). Stained cells were fixed with 1× Intracellular (IC) fixation buffer (Invitrogen, CA, USA) for 30 min on ice. Samples were acquired on a LSRFortessa flow cytometer (BD Biosciences, CA, USA) and data were analyzed with FlowJo software version 10.4.0 (BD Biosciences, CA, USA).

### 2.11 Analysis of alternative splicing (AS) events

Data on alternative splicing in NSCLC (LUAD and LUSC) were obtained from the online database of TCGA SpliceSeq. The Percent Spliced In (PSI) value was calculated to quantify the rate of every splicing event, and we included AS events where the percentage of samples with PSI values was over 75%. Seven types of alternative splicing were identified: Alternate Promoter (AP), Alternate Terminator (AT), Exon Skip (ES), Retained Intron (RI), Alternate Acceptor site (AA), Alternate Donor site (AD) and Mutually Exclusive Exons (ME). Differentially expressed AS events (DEAS) analysis was performed using Wilcoxon rank-sum test to compare the AS events from two groups. AS events with |log_2_FC| > 1 and *P* < 0.05 were considered as significantly changed AS events.

### 2.12 Risk score construction

Three genes with the highest kME were selected from each ICB-related module. Lasso-penalized Cox (Lasso-Cox) regression was performed using the R package glmnet (version 4.1-3) based on all genes to create a risk score for ICB responsiveness (34). We performed 5-fold cross-validation using the function ‘cv.glmnet’ to get the λ.min value, which is the optimal penalization coefficient (λ) value. The risk score was calculated as follows: risk score = sum (mRNA expression level of the screened genes × corresponding beta coefficient). We validated the established risk score using Kaplan-Meier analysis with the GLCI cohort and KM-plotter pan-cancer cohort. The time-dependent receiver operating characteristic (ROC) curve for survival data was analyzed using the R package survivalROC (version 1.0.3.1) at indicated time points. According to the risk score, samples were separated into high- and low-risk groups.

### 2.13 Tumor microenvironment deconvolution

We used multiple methods to deconvolute the TME composition of TCGA NSCLC samples. The estimate score was calculated using the R package estimate (version 1.0.13) to infer the stromal and immune composition in each sample (35). We used the CIBERSORTx online tool (https://cibersortx.stanford.edu/) with LM22 profile and a scRNA-seq profile for immune cell deconvolution (36). To further dissect the immune cell subsets in NSCLC samples, we customized the scRNA-seq profile based on the Bernard_Thienpont dataset (37–39). We also used the TIMER2.0 online tool (http://timer.cistrome.org/) to perform the deconvolution analyses with TIMER, quanTIseq, and xCell (40–42).

### 2.14 Somatic variant analysis

Somatic variant results from MuTect2 pipeline were used as input for the R package maftools (version 2.10.5) (43). We included clinically relevant frequently mutated genes for comparisons between high- and low-risk groups, and we drew plots via the maftools functions ‘coOncoplot’ and ‘coBarplot’. TMB was calculated using the maftools function ‘tmb’.

### 2.15 Statistical analysis

R software (version 4.1.2) was used for all statistical analysis. The Wilcoxon rank-sum test was used where indicated. The log-rank test was used to estimate the difference in Kaplan-Meier survival analysis. A *P* value < 0.05 was considered statistically significant (*: *P* < 0.05, **: *P* < 0.01, ***: *P* < 0.001, ****: *P* < 0.0001).

## 3 Results

### 3.1 Identification of co-expression modules related to ICB responsiveness in NSCLC

**Figure 1:**
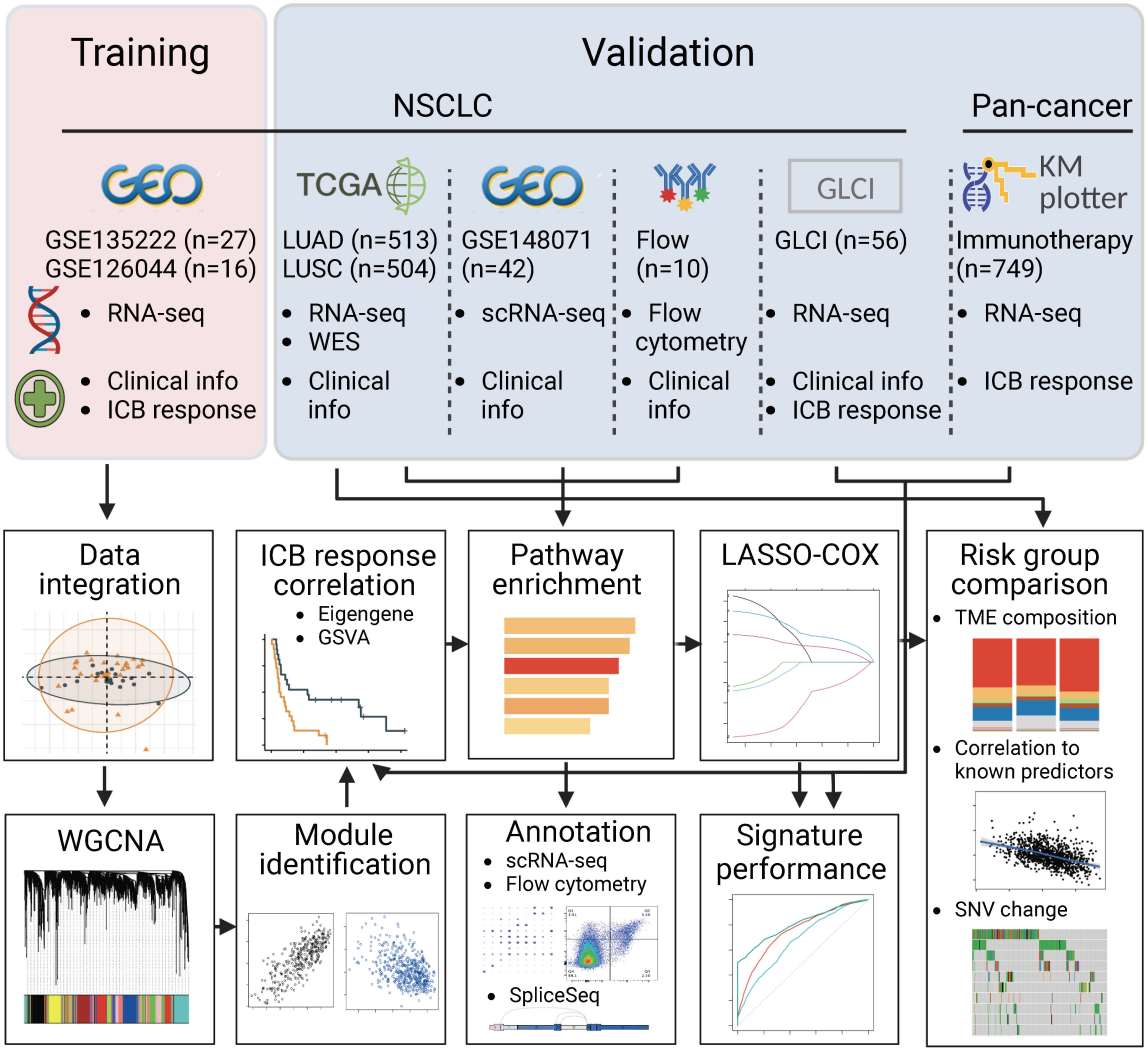
Flowchart of the study. In brief, we integrated two RNA sequencing datasets of NSCLC, GSE135222 and GSE126044, where samples were taken before PD-(L)1 blockade treatment. We used weighted gene co-expression network analysis and correlation analysis to identify modules related to PD-(L)1 responsiveness. We then characterized the biological function of the PD-(L)1-relevant modules using TCGA-NSCLC datasets, single-cell RNA sequencing data from GSE148071, and flow cytometry data analyzed in-house. Next, we constructed a risk score using Lasso-COX regression that could optimally predict PD-(L)1 responsiveness. We validated the risk score in our PD-(L)1-treated NSCLC cohort at the Guangdong Lung Cancer Institute (GLCI) and a pan-cancer immunotherapy cohort using the KM-plotter database. We compared the composition of the tumor microenvironment and mutational changes between the groups categorized by the risk score. Additionally, we correlated the association between the risk score and previously reported predictors to reveal potential mechanisms.

**Figure 2:**
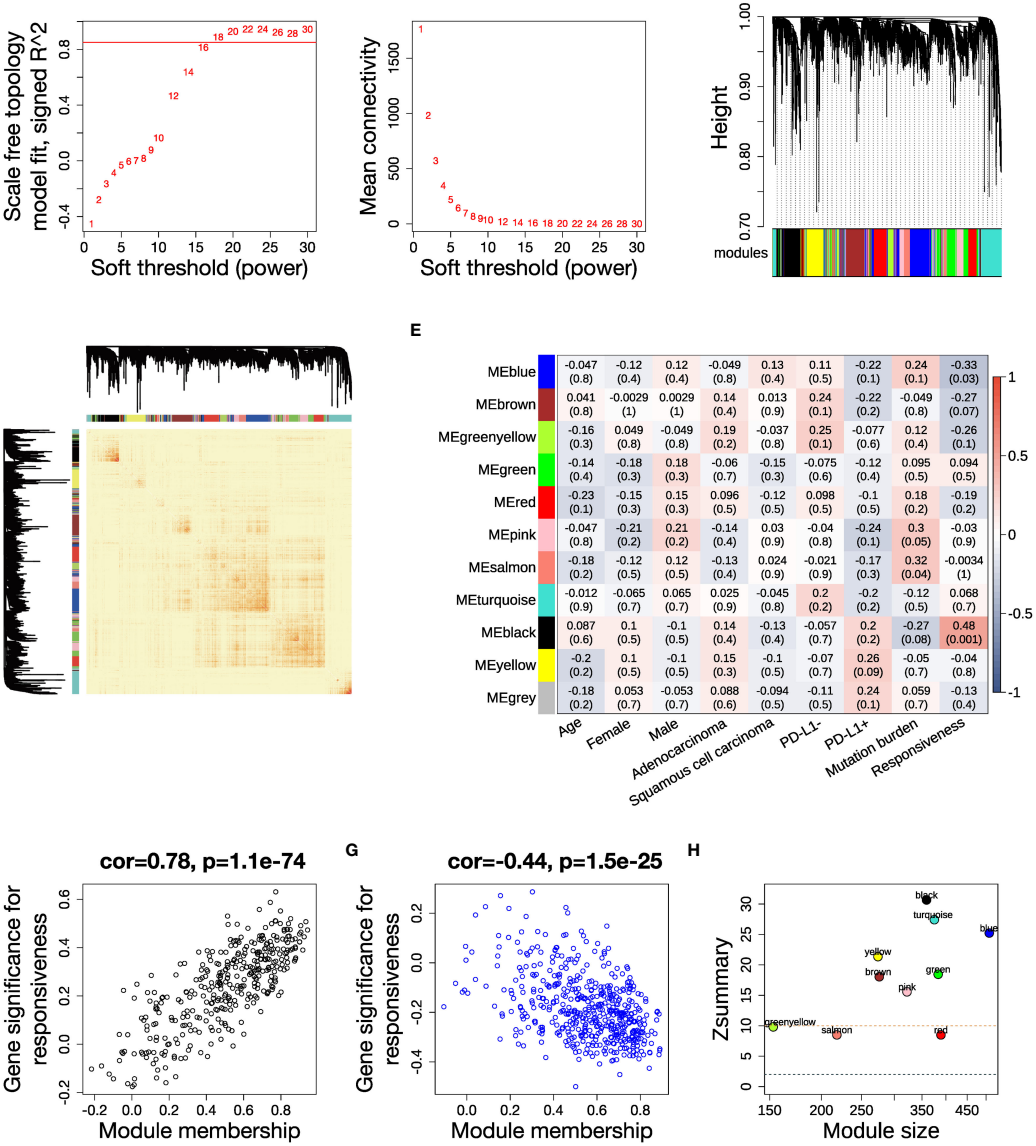
WGCNA analysis and identification of key modules related to ICB responsiveness. **(A)** Analysis of scale independence for a range of soft threshold powers with networkType set as “signed”. The red line represents the cutoff scale free topology index R^2^ = 0.85. **(B)** Analysis of mean connectivity for a range of soft threshold powers. **(C)** Cluster dendrogram of the 3316 genes with 11 modules labeled by colors. **(D)** Heatmap of the topological overlap matrix (TOM) displays the co-expression pattern of the 11 modules. **(E)** Heatmap of the module-trait correlations. Every cell shows the Pearson correlation coefficient and *P* value. **(F, G)** Scatter plots illustrating the correlations between module membership (kME) and gene significance for ICB responsiveness in the **(F)** black module and **(G)** blue module. **(H)** Analysis of module preservation in the TCGA NSCLC dataset. The Zsummary value represents the module preservation index, with higher values indicating stronger evidence of module preservation. A Zsummary > 10 (red dotted line) means strong evidence of module preservation, while a value < 2 (blue dotted line) means no evidence.

To fully characterize the transcriptomic profiles related to ICB responsiveness in NSCLC, we conducted the study as described in **Figure 1**. We integrated pre-treatment RNA-seq data from two public datasets, checking and correcting for batch effects (**Supplementary Figures 1A, B** **)**. We selected 3316 genes with high variance and retained all 43 samples after quality control (**Supplementary Figure 1C**). We set the soft thresholding power β to 18 as the smallest value to achieve scale free topology, resulting in a scale free topology index R^2^ of 0.89 and mean connectivity of 5.80 (**Figures 2A, B** **)**. 11 modules were eventually detected as illustrated by the cluster dendrogram (**Figure 2C**) and TOM heatmap (**Figure 2D**).

In order to elucidate the clinical association of the modules, we calculated the Pearson correlation between each clinical trait and module eigengenes, which represent a weighted average expression of each module (**Figure 2E**). The black module had a positive correlation, while the blue module had a negative correlation to anti-PD-(L)1 responsiveness (**Supplementary Table 5**). The relevance of assigned genes in the two modules to ICB responsiveness was verified by the module membership to gene significance correlation plots (**Figures 2F, G** **)**. We analyzed the preservation of the identified modules in the TCGA NSCLC dataset, which has a large sample size. The high Zsummary and low medianRank of both black and blue modules suggested they were strongly preserved in NSCLC (**Figure 2H**, **Supplementary Figure 1F**).

### 3.2 The black module has a positive correlation with ICB responsiveness

**Figure 3:**
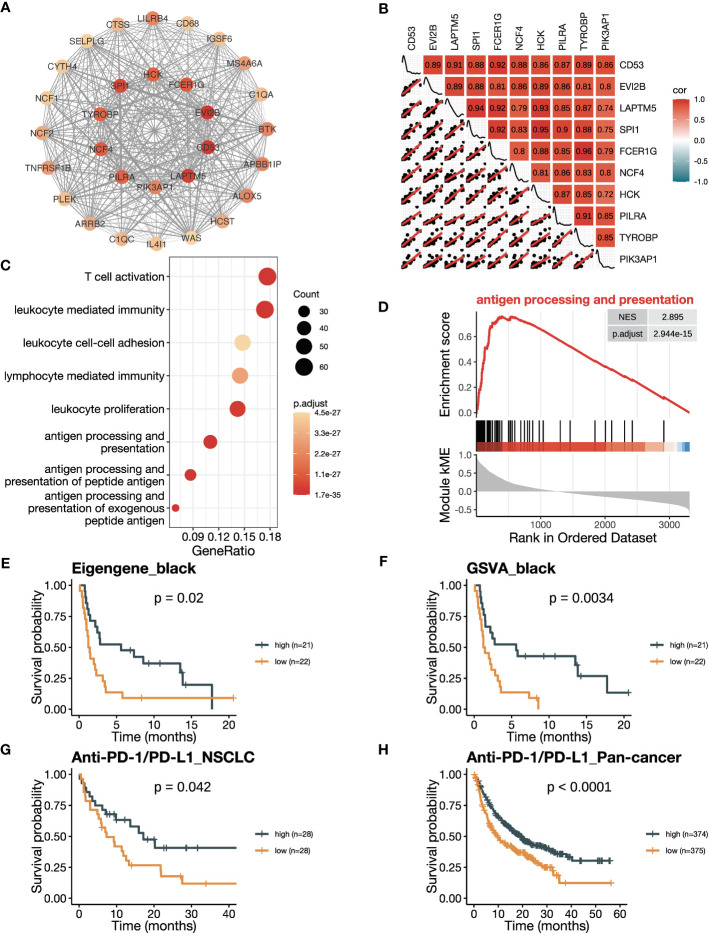
The black module is positively related to ICB responsiveness. **(A)** Co-expression network of the top 30 hub genes in the black module displayed using the software Cytoscape. The nodes are colored by the module membership (kME) and the edge thickness is proportional to the gene pair Pearson correlation coefficients. **(B)** Plot showing the correlation matrix among the top 10 hub genes in the black module. The upper triangle represents a heatmap of the Pearson correlation coefficients, and the lower triangle represents multiple scatter plots with a fitted regression line. **(C)** Gene ontology (GO) enrichment analysis shows the top eight enriched pathways in the black module. **(D)** Gene set enrichment analysis (GSEA) plot against GO demonstrates the enrichment of antigen processing and presentation (GO:0019882) in the black module. **(E)** Kaplan-Meier curve of PFS comparing patients with high eigengene of the black module to those with low eigengene in the training cohort (n=43). **(F)** Kaplan-Meier curve of PFS comparing patients with high GSVA scores of the black module (GSVA_black) to those with low scores in the training cohort (n=43). **(G, H)** Kaplan-Meier curve of OS comparing patients with high black module scores to those with low scores in **(G)** the NSCLC validation cohort in GLCI (n=56) and **(H)** the pan-cancer cohort from KM-plotter database (n=749). In the NSCLC cohort, black module scores were calculated using GSVA algorithm, while in the pan-cancer cohort, they were calculated using the mean expression of the top 10 hub genes due to limitation of the KM-plotter online database. The *P* values in **(E-H)** were derived from log-rank tests.

Firstly, we conducted an in-depth study of the black module to investigate its role in ICB responsiveness. Top 30 genes ranked by kME in the black modules were analyzed using the software Cytoscape, which revealed a co-expression network (**Figure 3A**). We further verified the co-expression pattern among the top 10 genes by heatmap and scatter plots (**Figure 3B**). To account for the confounding factor of tumor purity, we performed a purity adjustment, and the co-expression pattern was preserved (**Supplementary Figure 2A**). The top 10 genes were consistently downregulated in tumor versus normal tissues (**Supplementary Figure 2B**). Pathway enrichment analysis of the genes in the black module revealed T cell activation and antigen processing and presentation pathways, suggesting a potential mechanism for the module’s relation to ICB responsiveness (**Figure 3C**; **Supplementary Figure 2C**). The GSEA analysis also showed an enrichment of antigen processing and presentation and T cell activation pathways (**Figure 3D**; **Supplementary Figures 2D-F**). To confirm the positive correlation of the black module with ICB responsiveness, we performed Kaplan-Meier analysis in the training dataset. We found that a high eigengene of black module demonstrated an improvement of PFS after ICB therapy (**Figure 3E**). Furthermore, patients with high GSVA scores of the black module (GSVA_black) had better PFS (**Figure 3F**). We validated the OS benefits from ICB therapy in patients with high GSVA_black scores in cohorts of NSCLC in GLCI and pan-cancer from KM-plotter (**Figures 3G, H** **)**.

### 3.3 Genes of the black module show preferable expression in myeloid cells

**Figure 4:**
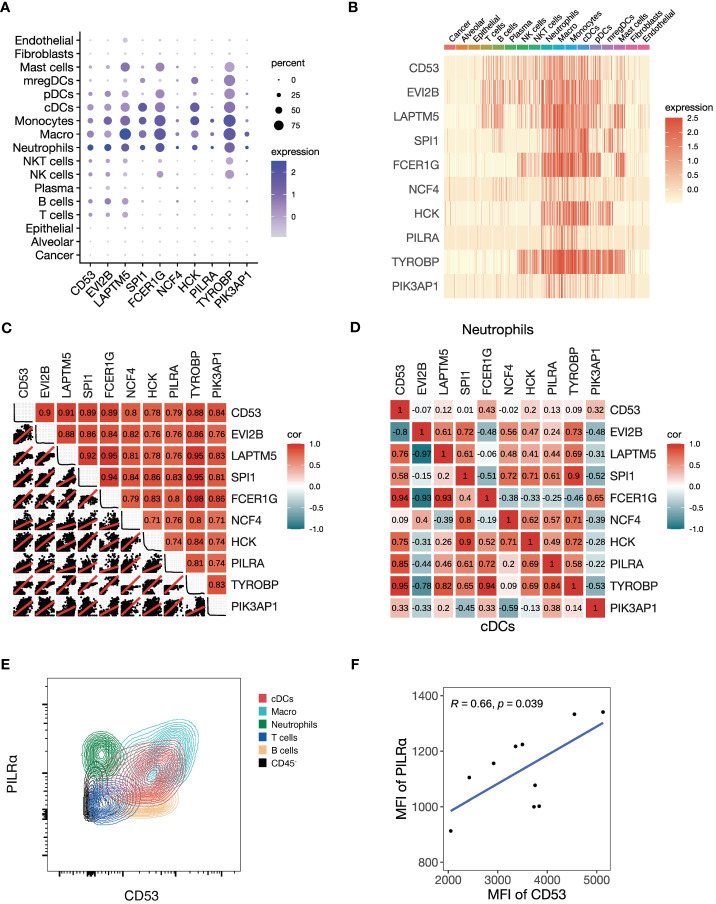
Preferable expression of the black module genes in myeloid cells. **(A-D)** We investigate the single-cell level RNA expression of the black module genes based on the scRNA-seq data of NSCLC (GSE148071). **(A)** Dot plot of all the cell types showing the expression of the top 10 hub genes in the black module. **(B)** Heatmap of all the cell types showing the expression of the top 10 hub genes in the black module. **(C)** Plot showing the correlation matrix of all the cell types pooled among the top 10 hub genes in the black module. The upper triangle represents a heatmap of the Pearson correlation coefficients, and the lower triangle represents multiple scatter plots with a fitted regression line. **(D)** Heatmap showing the correlation matrix of conventional dendritic cells (cDCs) and neutrophils among the top 10 hub genes in the black module. The upper triangle panel represents neutrophils and the lower triangle panel represents cDCs. **(E, F)** We conducted flow cytometry to explore the black module at the protein level. **(E)** Representative contour plot of flow cytometry comparing the fluorescent intensities of PILRα and CD53, which are the two representative hub genes of the black module, among different cell types. **(F)** Scatter plot showing the Pearson correlation between mean fluorescent intensities (MFI) of PILRα and CD53 in cDCs.

Given the black module’s association with T cell activation and antigen presentation, we explored the cellular expression of its assigned genes using the NSCLC scRNA-seq data. We found that the top 10 genes ranked by kME were predominantly expressed in immune cells, but not in cancer cells, alveolar cells, epithelial cells, fibroblasts and endothelial cells (**Figures 4A, B** **)**. Among immune cells, myeloid cells showed the highest expression of the module genes, including neutrophils, macrophages, monocytes, conventional dendritic cells (cDCs) and plasmacytoid dendritic cells (pDCs). Using a metacell expression matrix, we analyzed the correlations among the 10 genes based on all the cell types and found strong correlations among each gene pair (**Figure 4C**). It indicates that the specific expression of the black module genes in myeloid cells largely contributes to its co-expression pattern (**Supplementary Figure 3B**). In addition, the positive correlations of some gene pairs were observed in the neutrophils and cDCs (**Figure 4D**). To confirm the co-expression pattern of the black module genes at protein level, we performed flow cytometry analysis on NSCLC samples. We selected two representative genes *CD53* and *PILRA* for flow cytometry analysis, because they were among the hub genes of the black module and had commercially available fluorochrome-conjugated antibodies. We found that both CD53 and PILRα had a higher expression in macrophages, cDCs and neutrophils other than T cells, B cells and non-immune CD45^-^ cells (**Figure 4E**; **Supplementary Figures 3D, E** **)**. Moreover, the mean fluorescent intensities (MFI) of CD53 and PILRα were positively correlated in cDCs (**Figure 4F**). Our findings suggest that the black module genes show preferable expression in myeloid cells, which may contribute to their association with ICB responsiveness.

### 3.4 The blue module has a negative correlation with ICB responsiveness

**Figure 5:**
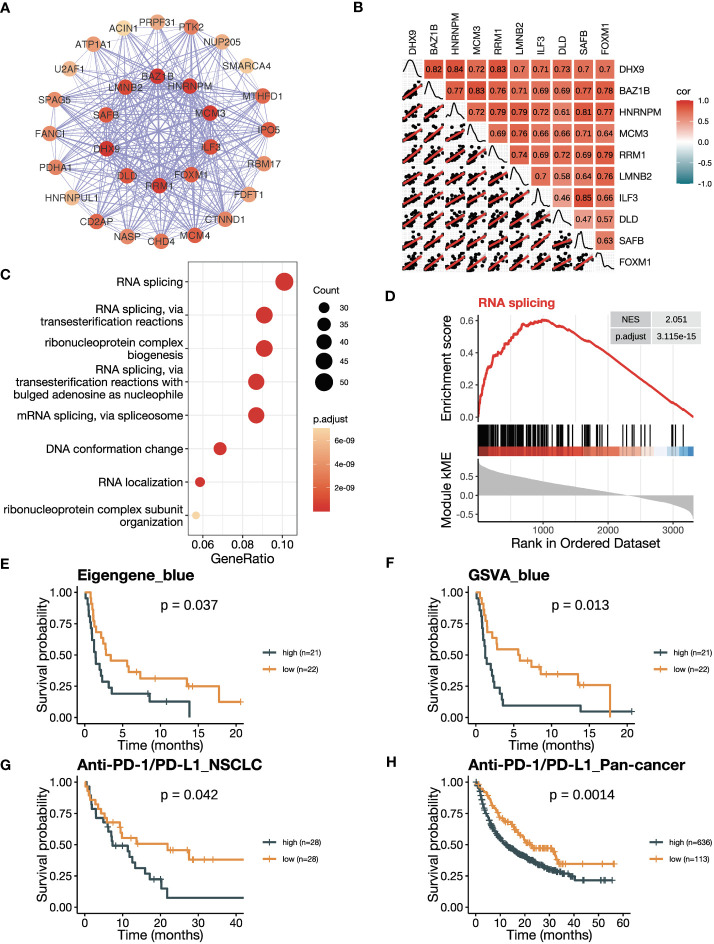
The blue module is negatively related to ICB responsiveness. **(A)** Co-expression network of the top 30 hub genes in the blue module displayed using the software Cytoscape. The nodes are colored by the module membership (kME) and the edge thickness is proportional to the gene pair Pearson correlation coefficients. **(B)** Plot showing the correlation matrix among the top 10 hub genes in the blue module. The upper triangle represents a heatmap of the Pearson correlation coefficients, and the lower triangle represents multiple scatter plots with a fitted regression line. **(C)** Gene ontology (GO) enrichment analysis shows the top eight enriched pathways in the blue module. **(D)** Gene set enrichment analysis (GSEA) plot against GO demonstrates the enrichment of RNA splicing (GO:0008380) in the blue module. **(E)** Kaplan-Meier curve of PFS comparing patients with high eigengene of blue module to those with low eigengene in the training cohort (n=43). **(F)** Kaplan-Meier curve of PFS comparing patients with high GSVA scores of the blue module (GSVA_blue) to those with low scores in the training cohort (n=43). **(G, H)** Kaplan-Meier curve of OS comparing patients with high blue module scores to those with low scores in **(G)** the NSCLC validation cohort in GLCI (n=56) and **(H)** the pan-cancer cohort from KM-plotter database (n=749). In the NSCLC cohort, blue module scores were calculated using GSVA algorithm, while in the pan-cancer cohort, they were calculated using the mean expression of the top 10 hub genes due to limitation of the KM-plotter online database. The *P* values in **(E-H)** were derived from log-rank tests.

We then explored the correlation between the blue module with ICB responsiveness. A co-expression network was visualized using the top 30 hub genes ranked by kME in the blue modules (**Figure 5A**). The heatmap and scatter plots illustrated that the top 10 hub genes were positively related to each other (**Figure 5B**). After adjusting for tumor purity, the co-expression pattern was preserved (**Supplementary Figure 4A**). Notably, the top 10 genes were mostly upregulated in tumor versus normal tissues (**Supplementary Figure 4B**). The blue module was enriched in pathways related to RNA splicing (**Figure 5C**; **Supplementary Figure 4C**), which was verified by GSEA analysis (**Figure 5D**; **Supplementary Figures 4D–F**). To confirm the negative correlation of blue module with ICB responsiveness, we performed Kaplan-Meier analysis and found that the patients with a high eigengene of blue module had worse PFS after ICB therapy (**Figure 5E**). Furthermore, patients with high GSVA scores of the blue module (GSVA_Blue) had shorter PFS (**Figure 5F**). We validated the results in the cohorts of NSCLC in GLCI and pan-cancer from KM-plotter, where the OS was decreased in patients with high GSVA_Blue scores (**Figures 5G, H** **)**.

### 3.5 Genes of the blue module are associated with RNA alternative splicing

**Figure 6:**
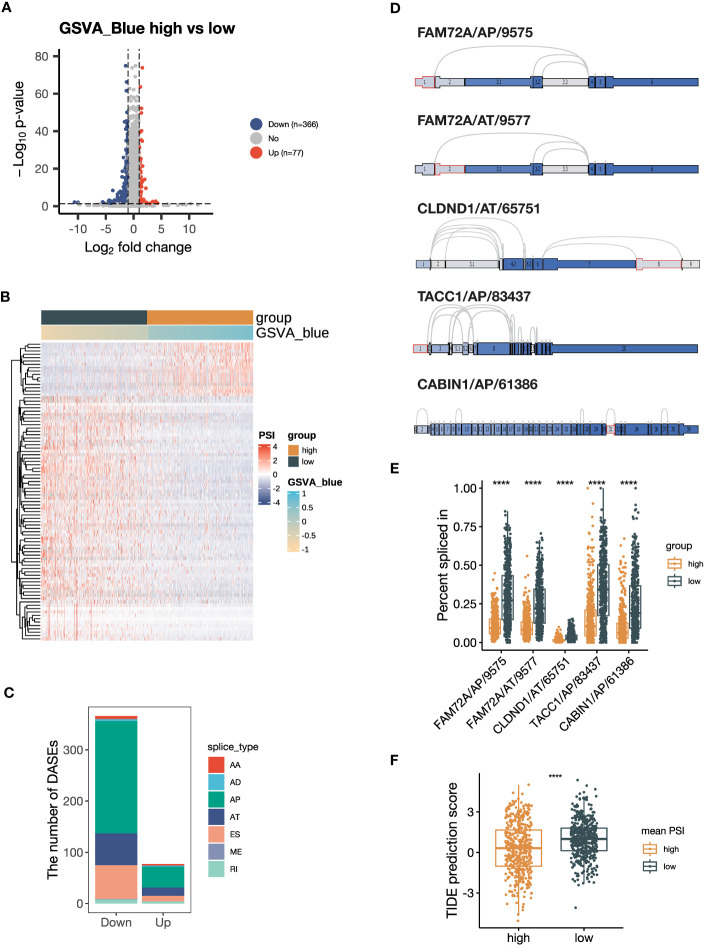
Alternative splicing (AS) events are downregulated in samples with high expression of blue module genes. **(A)** Volcano plot showing the differentially expressed AS events (DEASs) comparing the group with high GSVA scores of the blue module (GSVA_blue) versus the low GSVA_blue group. AS events with |log_2_FC| > 1 and *P* < 0.05 are considered significantly dysregulated. **(B)** Heatmap showing the scaled percent spliced in (PSI) values of the top 20% of DEASs with the lowest *P* values. **(C)** Stacked bar plot showing the number of seven types of DEASs in the high GSVA_blue group. **(D)** Splice graph illustrating the top 5 most downregulated AS events. The selected AS events are outlined by red, with exons shaded by expression level and splice paths connected by arcs. **(E)** Box plot comparing the PSI values of the top 5 most downregulated AS events between the high and low GSVA_blue groups. **(F)** Box plot comparing Tumor Immune Dysfunction and Exclusion (TIDE) prediction scores between the groups categorized by the average PSI values of the top 5 most downregulated AS events. The group with low TIDE prediction scores is more likely to be ICB responsive. The *P* values in **(E, F)** were derived from Wilcoxon rank-sum test. ****:*P* < 0.0001. In box plots, the central line is the median, and the limits are the upper and lower quartiles. AA, Alternate Acceptor site; AD, Alternate Donor site; AP, Alternate Promoter; AT, Alternate Terminator; ES, Exon Skip; ME, Mutually Exclusive Exons; RI, Retained Intron.

As indicated by the pathway enrichment analysis, the blue module was associated with RNA alternative splicing. To investigate this further, we performed DASE analysis based on the NSCLC dataset from TCGA SpliceSeq database. Our results revealed that the group with a high GSVA_Blue score had a preference for downregulated AS events, with 366 significantly downregulated AS events and 77 significantly upregulated AS events (**Figure 6A**). The heatmap also demonstrated that the GSVA_Blue score was relevant to the dysregulated AS events (**Figure 6B**). The major types of downregulated AS were AP, AT and ES (**Figure 6C**). As illustrated by the UpSet plot, most genes only contained one type of AS (**Supplementary Figure 5A**). The top 5 most downregulated AS events were FAM72A/AP/9575, FAM72A/AT/9577, CLDND1/65751/AT, TACC1/AP/83437 and CABIN1/AP/61386 (**Figures 6D, E** **)**. We calculated the average PSI values of the top 5 AS events and found that the group defined by a high average value was more likely to be ICB responsive, as suggested by the lower TIDE prediction score (**Figure 6F**) (44). Our findings suggest that the genes of the blue module are associated with RNA alternative splicing, and that downregulated AS events may contribute to a negative correlation with ICB responsiveness.

### 3.6 Integrative risk score of the black and blue modules are associated with ICB responsiveness

**Figure 7:**
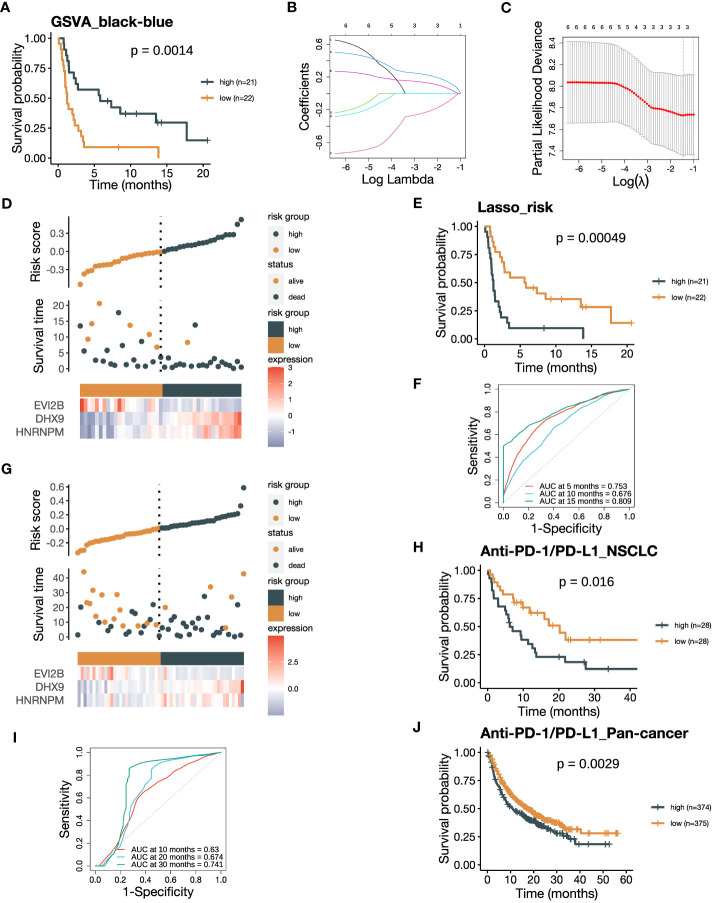
The risk score integrating the black and blue modules negatively associates with ICB responsiveness. **(A)** Kaplan-Meier curve of PFS comparing patients with high GSVA_black-blue scores to those with low scores in the training cohort (n=43). The GSVA_black-blue score was calculated by subtracting the GSVA_blue score of each sample from the corresponding GSVA_black score. **(B)** Lasso coefficient profiles of the six hub genes, each three with the highest module membership (kME) from the black and blue modules. **(C)** The optimal penalization coefficient (λ) was calculated using 5-fold cross-validation based on partial likelihood deviance, which yielded λ.min = 0.234. **(D)** The distribution of the PFS and the expression of three screened genes in the risk score based on the Lasso-COX model (training dataset). **(E)** Kaplan-Meier curve of PFS comparing patients with high risk scores to those with low scores from the training dataset (n=43). **(F)** The receiver operating characteristic (ROC) curve of PFS showing the area under the curve (AUC) of the risk score at 5, 10, 15 months in the training dataset. **(G)** The distribution of the OS and the expression of three screened genes in the risk score (GLCI validation dataset). **(H)** Kaplan-Meier curve of OS comparing patients with high risk scores to those with low scores from the GLCI validation dataset (n=56). **(I)** The ROC curve of OS showing the AUC of the risk score at 10, 20, 30 months in the GLCI validation dataset. **(J)** Kaplan-Meier curve of OS comparing pan-cancer patients with high risk scores to those with low scores from the KM-plotter validation dataset (n=749). The *P* values in **(A, E, H, J)** were derived from log-rank tests.

The contrary associations of the black and blue modules with ICB responsiveness indicated their complementary characteristics of predicting response to ICB therapy. Therefore, we attempted to develop a model that could predict ICB responsiveness based on these modules. We firstly subtract the GSVA_blue score from the corresponding GSVA_black score, resulting in a new GSVA_black-blue score. The GSVA_black-blue score effectively stratified PFS between patients with high and low scores in the training cohort (**Figure 7A**). The favorable OS in the group with high GSVA_black-blue score was also validated in the NSCLC cohort in GLCI (**Supplementary Figure 6A**). To create a signature predicting ICB responsiveness with a limited number of genes, we performed Lasso-COX regression analysis on the six hub genes, each with the three highest kME from the two modules (**Figure 7B**). The algorithm screened out three genes, *EVI2B*, *DHX9* and *HNRNPM*, and we built the Lasso-COX model with λ.min = 0.234 as the optimal λ coefficient (**Figure 7C**). The risk score derived from the model was (-0.083 × expression level of *EVI2B*) + (0.126 × expression level of *DHX9*) + (0.050 × expression level of *HNRNPM*). Higher risk scores indicated the patients were at higher risk of poor clinical responses to ICB therapy with shorter survival (**Figure 7D**). Also, the risk score could significantly divide the training cohort into two groups, with better PFS in the low-risk group (**Figure 7E**). The ROC curve of the training cohort demonstrated that the areas under the curve (AUCs) in predicting survival benefits after ICB treatment at 5, 10, and 15 months were 0.753, 0.676, and 0.809 (**Figure 7F**). We validated the risk score in the NSCLC cohort in GLCI, where a lower risk of poor ICB response was observed in the low-risk group (**Figure 7G**). The Kaplan-Meier analysis of OS in the NSCLC validation cohort also verified the predictive value of the risk score in recognizing patients who failed to response to ICB therapy, yielding the AUCs at 5, 10, and 15 months being 0.630, 0.674, and 0.741 (**Figures 7H, I** **)**. Finally, we validated the risk score in the pan-cancer cohort from the KM-plotter database, where the OS of ICB-treated patients with low risk scores was significantly prolonged (**Figure 7J**). Our results suggest that the integrative risk score of the black and blue modules is associated with ICB responsiveness, and the three-gene signature may serve as a useful tool for predicting response to ICB therapy.

### 3.7 The low-risk group of NSCLC samples shows an immune-active TME

**Figure 8:**
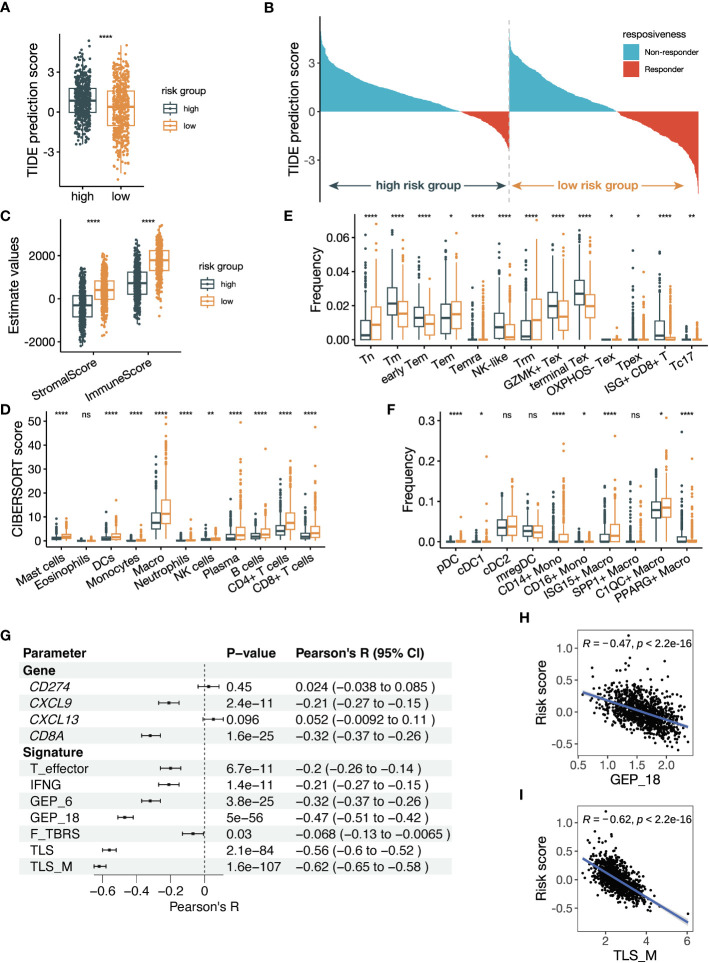
The risk score is related to tumor microenvironment alteration and previously established parameters predicting ICB responsiveness. **(A)** Box plot comparing Tumor Immune Dysfunction and Exclusion (TIDE) prediction scores between groups with high and low risk scores. The group with low TIDE prediction scores is more likely to be ICB responsive. **(B)** Waterfall plot of TIDE prediction scores colored by ICB responsiveness. The left half represents patients with high risk scores, and the right half represents patients with low risk scores. **(C)** Box plot showing the StromalScore and ImmuneScore calculated by the estimate algorithm between high- and low-risk groups. **(D)** Box plot showing the cell abundance of major immune cells between high- and low-risk groups using the CIBERSORTx tool run in absolute mode. The signature matrix used is the LM22 profile. **(E, F)** Box plot showing the cell frequencies of **(E)** CD8^+^ T cell subsets and **(F)** myeloid cell subsets between high- and low-risk groups using the CIBERSORTx tool run in relative mode. The signature matrix used is customized based on the Bernard_Thienpont NSCLC scRNA-seq. The mean frequencies of Tpex in the high- and low-risk groups are 0.15% and 0.17%, respectively. **(G)** Forest plot showing the Pearson correlation between previously reported parameters regarding ICB prediction and the risk score. **(H, I)** Scatter plots illustrating the correlations of **(H)** GEP_18 and **(I)** tertiary lymphoid structure in melanoma (TLS_M) signatures versus the risk score. All the data in **(A–I)** were analyzed using the TCGA NSCLC dataset. The *P* values in **(A, C–F)** were derived from Wilcoxon rank-sum test. ns: not significant, *:*P* < 0.05, **:*P* < 0.01, ****:*P* < 0.0001. In box plots, the central line is the median, and the limits are the upper and lower quartiles.

To investigate the underlying causes of the associations with ICB therapy, we compare the TME composition between the high-risk and low-risk groups defined by the risk score. We analyzed the TCGA NSCLC dataset, considering the large sample size and data comprehensiveness. Firstly, we utilized the TIDE algorithm to predict the ICB responsiveness of TCGA patients, and the lower TIDE prediction score in the low-risk group indicated a higher likelihood of patients to benefit from ICB treatment (**Figure 8A**). The dichotomous results from TIDE prediction also demonstrated an obvious shift of patients with low risk scores towards ICB responders (**Figure 8B**). Next, we adopted the estimate score to quantify the cellular composition, and the higher StromalScore and ImmuneScore suggested a stromal- and immune-enriched TME (**Figure 8C**). To further dissect the immune microenvironment, we performed CIBERSORTx analysis using the LM22 signature. Most immune cell subsets were significantly more infiltrated in the low-risk group, including the myeloid cells where the black module genes were expressed (**Figure 8D**). In addition, we performed TIMER, quanTIseq, and xCell analyses, and the results also showed more immune infiltration in the low-risk group (**Supplementary Figures 7B-D**). Our findings suggest that the low-risk group of NSCLC samples exhibit an immune-active TME, which may explain their increased likelihood of benefiting from ICB treatment.

Next, we delved deeper into comparing T-cell and myeloid cell subsets, which have been proved to play a large part in anti-tumor immunity. We used the NSCLC scRNA-seq data to create a signature for the estimation of T cell and myeloid cells subsets (**Supplementary Figure 7E**). CD8^+^ T cells are well-known immune cell types that determines ICB responsiveness. Tumor samples from ICB responders were found to have a low frequency of terminal exhausted CD8^+^ T cells (Tex) and high frequency of tissue-resident memory CD8^+^ T cells (Trm) (38), which was consistent with our result (**Figure 8E**). In addition, the estimated frequency of precursor exhausted CD8^+^ T cells (Tpex) and Type 17 CD8^+^ T cells (Tc17) was increased in the low-risk group as well. Tpex, marked by TCF-1^+^, was considered to generate effector CD8^+^ T cells in response to ICB therapy (45). The relevance of CD4^+^ T cells with ICB responsiveness was less established. We observed a decreased frequency of classic follicular helper T (Tfh) cells and an increased frequency of *IFNG^+^* Tfh/T helper 1 (Th1) cells in the low-risk group (**Supplementary Figure 7F**). Frequencies of regulatory T (Treg) cells were also upregulated except *TNFRSF9^-^* Treg cells, which was a resting subtype of Treg cells. Our findings suggest that the low-risk group of NSCLC samples have a more favorable T-cell composition to mediate the anti-PD-(L)1 treatment.

Myeloid cells can modulate the anti-tumor immune reaction directly or indirectly through regulating T cells. Among the dendritic cells subsets, frequencies of type 1 cDCs (cDC1s) and pDCs were significantly increased in the low-risk group (**Figure 8F**). The frequency of classical CD14^+^ monocytes was upregulated while that of non-classical CD16^+^ monocytes was downregulated in the low-risk group. Moreover, *C1QC^+^* macrophages and *ISG15^+^* macrophages had higher frequencies, while *PPARG^+^* lung-resident alveolar macrophages had a lower frequency in the low-risk group. These findings suggest that alterations of myeloid cell subsets in the low-risk group may play a role in modulating the anti-tumor immune response during ICB therapy.

We compared the mutational landscape of TCGA-LUAD and LUSC cohorts between the two risk groups. Among the most common somatic mutations of LUAD, *TP53*, *KRAS*, *KEAP1* and *STK11* were less mutated in the low-risk group (**Supplementary Figures 8A, B** **)**. The frequencies of *TP53* and *CDKN2A* were decreased in the low-risk group of LUSC, while that of *FAT1* was increased (**Supplementary Figures 8C, D** **)**. The TMB of LUAD and LUSC had a decrease or a decrease trend in the low-risk groups (**Supplementary Figures 8E, F** **)**. Overall, the mutational changes in the low-risk groups were not consistent with the trend towards a better response to ICB treatment reported by the other studies. Therefore, the mutational profiles are less important in evaluating the significance of the risk score.

### 3.8 The correlation of the risk score with established parameters that predicts response to ICB therapy

We investigated the correlation between our risk score and established parameters that predict anti-PD-(L)1 responsiveness. We computed the parameters reported by previous publication, including single genes and gene signatures, and performed correlation analysis with our risk score. The single genes included were *CD274* (PD-L1), *CXCL9* (46), *CXCL13* (47) and *CD8A* (48). We also included signatures of T effector, IFNG, 6-gene GEP, 18-gene GEP, pan-fibroblast TGFβ response signature (F-TBRS) (49), TLS and TLS in melanoma (TLS_M) (50). Multiple parameters were found to be significantly related to the risk score (**Figure 8G**). *CD8A* and *CXCL9* were significantly associated with the risk score, while the signatures of TLS/TLS_M and GEP_18 were the most significant ones correlated with the risk score (**Figure 8H, I**; **Supplementary Figures 7G-L**). These findings suggest that our risk score is strongly correlated with established parameters that predict ICB responsiveness, especially gene signatures of TLS/TLS_M and GEP_18.

## 4 Discussion

In this study, we leverage WGCNA analysis to comprehensively characterize the transcriptome of baseline NSCLC samples before anti-PD(L)1 therapy. We identified two co-expression modules related to ICB responsiveness that were preserved in NSCLC samples. The black module was positively correlated to ICB responsiveness and enriched in pathways of antigen processing and presentation and T cell activation. Data from scRNA-seq and flow cytometry revealed that the genes in the immune-related black module had a preferable expression pattern in myeloid cells. In addition, the blue module was negatively associated with ICB responsiveness, and samples with high expression of blue module genes tended to downregulate AS events. The downregulated AS events, mostly AP, AT and ES types, were positively associated with ICB responsiveness. We established a three-gene risk score using Lasso-COX regression analysis from the two ICB-related modules, and validated its predictive value for ICB therapy failure in a NSCLC dataset and a pan-cancer dataset. The risk groups defined by the risk score were compared to dissect the differences in the TME profiles. The low-risk group, which was more responsive to ICBs, was more stromal- and immune-infiltrated. Furthermore, the low-risk group had higher frequencies of Tpex, Tc17, pDCs and cDC1s, and featured as Tex^lo^ Trm^hi^, which could contribute to the superior responsiveness. We also found that the risk score had a significant correlation to the previously reported ICB-predictive parameters, especially the TLS_M and GEP_18 signatures, which partly accounted for its predictive value.

Previous studies have shown that there are no significant differences in response to anti-PD-(L)1 treatment in NSCLC patients based on race or age (51, 52). However, a large meta-analysis has illustrated that Asian patients experience greater benefits from anti-PD-(L)1 therapy compared to non-Asian patients (53). As our dataset primarily consists of Asian patients, further validation of our results in non-Asian NSCLC datasets is necessary. Research has shown that the effectiveness of anti-PD-(L)1 treatment may be influenced by gender (54, 55). Conforti et al. performed a meta-analysis of randomized clinical trials, and found that men with NSCLC experience a significantly greater benefit from ICB therapy compared to women, even in patients with high PD-L1 expression levels (56). However, our study demonstrates that the two ICB-related modules do not have any correlation with gender, as depicted in **Figure 2E**.

Several hub genes in the black modules, which was positively correlated with ICB responsiveness, have been studied and may shed light on the mechanism of ICB responsiveness. *CD53*, with the highest kME of the black module, is a member of tetraspanins. Dunlock et al. showed that *CD53* knockout mice experienced impaired tumor rejection due to the restrained T cell proliferation and activation, but did not thoroughly study the function of CD53 in myeloid cells (57). CD53-mediated anti-tumor immunity could be a factor that promotes the response to ICB treatment. Lysosomal-associated protein transmembrane 5 (LAPTM5) in macrophages acts a positive modulator to transmit inflammatory signaling and produces proinflammatory cytokines in return, such as TNF-α, and IL-12 (58). Due to the anti-tumor role of TNF-α and IL-12, the expression of *LAPTM5* in macrophages could be a potential mechanism to improve ICB efficacy. Zheng et al. reported that PILRα on myeloid cells interacts with CD8α to maintain CD8^+^ T cell quiescence (59). This PILRα-CD8α interaction could likely enhance the pools of naïve and memory CD8^+^ T cell and further maintain ICB therapy responsiveness.

Alternative splicing is a mechanism of a single gene to produce diverse transcripts and is dysregulated in multiple cancers (60). Splicing events of tumor-specific mRNA frequently introduces neoepitopes that can be presented by major histocompatibility complex class I (MHC-I) and subsequently recognized by T cells (61). Tumor-specific splicing events may serve as a predictive biomarker for ICB responsiveness. Compared to the neoantigens derived from mutations, splicing-derived neoantigens are more commonly detected and may become the ideal target for novel tumor immunotherapy (62, 63). *DHX9*, the top hub gene with the highest kME in the blue module, has been found to be relevant to defection of alternative splicing in tumor cells and promotes the formation of R-loop structures of nucleic acids (64). *HNRPM* belongs to the heterogeneous nuclear ribonucleoproteins (hnRNP) family and is able to modulate alternative splicing via exon skipping or exon inclusion (65, 66).

The risk score created by the Lasso-COX model identified NSCLC patients with low risk scores who may benefit from ICB therapy. The low-risk group was more immune-infiltrated, consistent with the immunologically hot tumor type (67). CD8^+^ T cells become exhausted with poor effector functions in cancer where antigen stimulation persists (68), while the CD8^+^ Trm cells are native tissue defenders with protective functions against tumor cells (69). A pan-cancer study identified a tumor type defined by a low frequency of terminal Tex and a high frequency of Trm, and this Tex^lo^ Trm^hi^ feature was associated with ICB responsiveness (38). Recent studies have shown that Tpex cells, defined as TCF1^+^PD-1^+^CD8^+^ T cells, can give rise to Tex cells and are believed as the key cell subset that responds to ICB therapy (45). Our estimated frequency of Tpex was relatively low, consistent with other reports, but its increase in the low-risk group is a potential cause of ICB responsiveness. Tc17, a CD8^+^ T cell subset producing IL-17, was also implicated as a player in the ICB treatment (38). The association of CD4^+^ T cells with ICB responsiveness is not well characterized, and our results regarding the alteration of Tfh and Treg cells in the low-risk group need further investigation.

Several types of myeloid cells were found to be different between the high- and low-risk groups. Patients with a high signature of dendritic cells in the pre-treatment samples were more likely to have ICB responses, suggesting that the anti-tumor impact of anti-PD-(L)1 is mediated by DCs (70). Blocking the PD-1/PD-L1 interaction between pDCs and effector cells abolished the immune suppression of pDCs on T cells and NK cells (71). Dahling et al. found that cDC1s could provide a niche to main Tpex cells and prevent their overactivation dependent on MHC-I interactions, and this shielding effect on Tpex was associated with ICB responsiveness (72). Macrophages have diverse subsets revealed by robust results of scRNA-seq and defined by marker genes as *SPP1^+^*, *C1QC^+^*, *PPARG^+^*, and *ISG15^+^* macrophages. *C1QC^+^* macrophages are mutually exclusive to *SPP1^+^* macrophages, and they co-expressed other C1q genes, *HLA-DR*, *APOE*, and *MRC1* (73). Interestingly, C1q genes and *APOE* were included in a TLS signature of renal cell cancer associated with better ICB response, indicating a potential role of *C1QC^+^* macrophages in helping to eliminate tumor cells in ICB therapy (74). *ISG15^+^* macrophages upregulated multiple interferon-induced genes and M1-like markers, but they are also suppressive through tryptophan degradation (75). Therefore, the anti-tumor role of *ISG15^+^* macrophages require further studies. PPARG^+^ macrophages are lung-resident alveolar macrophages, but their function in the TME is not well clarified yet.

The risk score is most closely related to the signature of TLS, providing an underlying mechanism of ICB responsiveness in the low-risk group. TLS is ectopic lymphoid aggregates in the tumor that feature B cells surrounded by T cells, similar to the secondary lymphoid organs. Multiple studies have demonstrated the value of TLS as a biomarker associated with benefits from ICB treatment in various types of tumors, including NSCLC, renal cell carcinoma, melanoma (50, 74, 76). The immune cell subsets changed in our study, such as the *C1QC^+^* macrophages discussed earlier, may also contribute to the composition of TLS.

In conclusion, our analysis revealed two ICB-related co-expression modules in baseline NSCLC samples prior to ICB therapy. The black module, which was positively associated with ICB responsiveness, had pathway enrichment of antigen processing and presentation and T cell activation, with its assigned genes mostly expressed in myeloid cells. The blue module had a negative correlation with ICB responsiveness and was associated with decreased alternative splicing events. A risk score constructed based on the two modules could be a surrogate marker to predict the risk for poor benefits from ICB treatment. Tumors with low risk scores were more immune-infiltrated. T cell composition changed in the low-risk group in favor of anti-tumor immunity, with increased Tpex, Trm and Tc17 and decreased terminal Tex. The higher frequencies of pDCs, cDC1s, *C1QC1^+^* macrophages and *ISG15^+^* macrophages in the low-risk group could also be potential mechanisms that promote response to ICB therapy. In addition, the strong correlation to TLS formation makes the risk score more robust. Our study provides a perceptive insight into the transcriptomic profile of NSCLC and a clinically translatable predictor for ICB responsiveness. However, further studies are needed to validate the results.

## Data availability statement

The datasets presented in this study can be found in online repositories. The names of the repository/repositories and accession number(s) can be found within the article/**Supplementary Materials**.

## Ethics statement

The studies involving human participants were reviewed and approved by The Ethics Committee of Ruijin Hospital, Shanghai Jiao Tong University School of Medicine. The patients/participants provided their written informed consent to participate in this study.

## Author contributions

YH, S-YL: Conceptualization, Data curation, Formal analysis, Investigation, Methodology, Writing - original draft. RJ, WM: Writing - Review & Editing. Y-LW: Data curation, Writing - Review & Editing. HL: Conceptualization, Resources, Funding acquisition, Project administration, Supervision, Writing - Review & Editing. All authors contributed to the article and approved the submitted version.
