# Supplementary material for: A risk score combining co-expression modules related to myeloid cells and alternative splicing associates with response to PD-1/PD-L1 blockade in non-small cell lung cancer
Source: Front Immunol. 2023 Jul 10;14:1178193. doi: 10.3389/fimmu.2023.1178193 (PMC10363729; doi:10.3389/fimmu.2023.1178193)
Supplement: Supplementary file 1 [file DataSheet_1.pdf]

Supplementary Material

A risk score combining co-expression modules related to myeloid cells and alternative splicing associates with response to PD-1/PD-L1 blockade in non-small cell lung cancer

Yichao Han[1†], Si-Yang Maggie Liu[2, 3†], Runsen Jin[1], Wangyang Meng[1], Yi-Long Wu[3*], Hecheng Li[1*]

*** Correspondence:** Hecheng Li: lihecheng2000@hotmail.com; Yi-Long Wu, syylwu@live.cn

1 Supplementary Figures

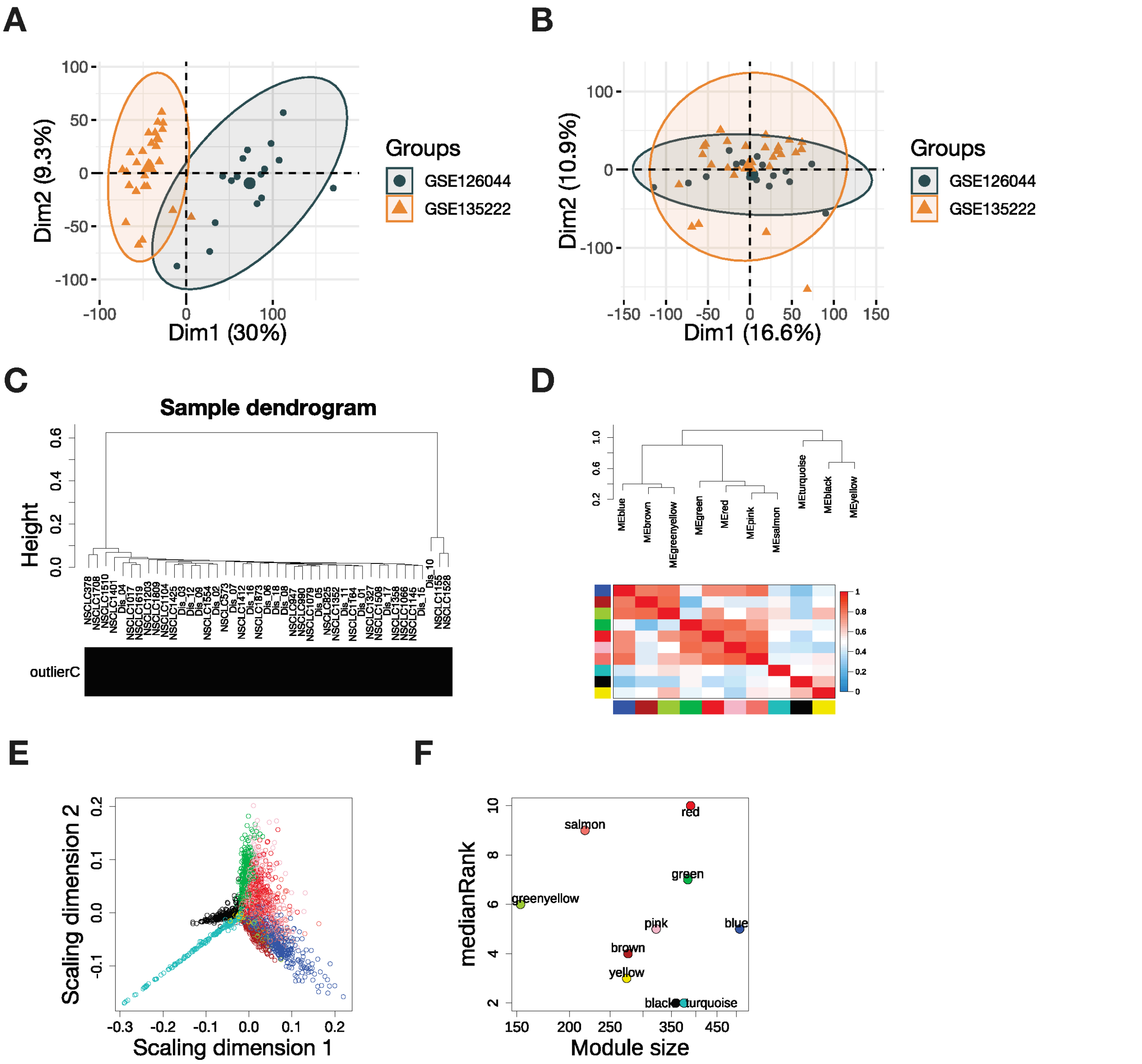

Supplementary Figure 1. Preprocessing of the input data for WGCNA analysis and module identification and preservation, related to Figure 2. **(A)** Principal component analysis (PCA) of RNA-seq data before batch effect correction. **(B)** PCA of RNA-seq data after batch effect correction. **(C)** Hierarchical clustering of samples demonstrates no outliers. **(D)** Correlation between identified modules. **(E)** Multidimensional scaling plot visualizes module correlation. **(F)** Analysis of module preservation in the TCGA NSCLC dataset. The lower the medianRank, the more preserved the module.

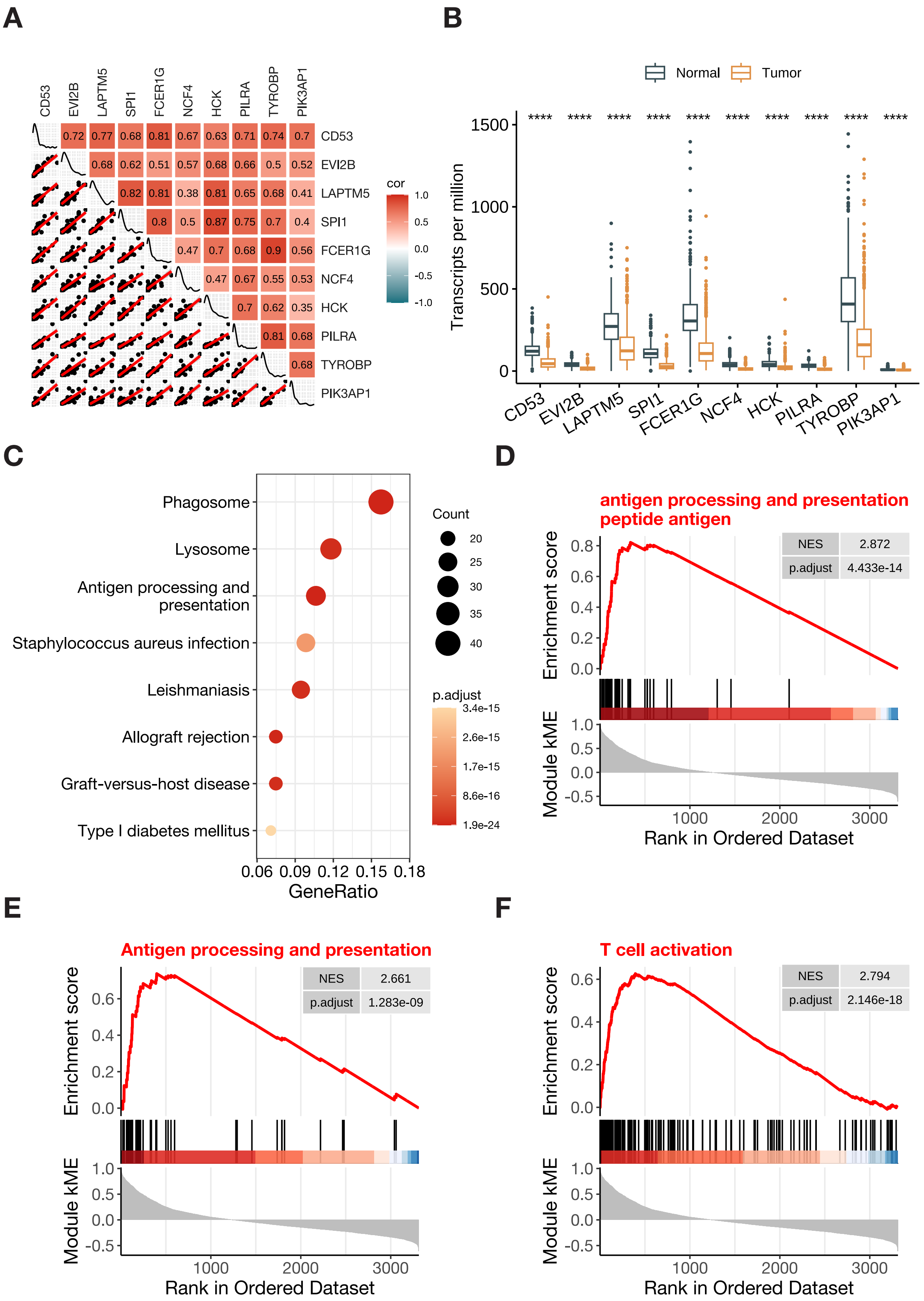
A
CD53
EVI2B
LAPTM5
SPI1
FCER1G
NCF4
HCK
PILRA
TYROBP
PIK3AP1
cor
1.0
0.5
0.0
-0.5
-1.0
B
Normal
Tumor
Transcripts per million
****
C
Phagosome
Lysosome
Antigen processing and presentation
Staphylococcus aureus infection
Leishmaniasis
Allograft rejection
Graft-versus-host disease
Type I diabetes mellitus
GeneRatio
Count
p.adjust
D
antigen processing and presentation peptide antigen
NES 2.872
p.adjust 4.433e-14
Enrichment score
Module kME
Rank in Ordered Dataset
E
Antigen processing and presentation
NES 2.661
p.adjust 1.283e-09
Enrichment score
Module kME
Rank in Ordered Dataset
F
T cell activation
NES 2.794
p.adjust 2.146e-18
Enrichment score
Module kME
Rank in Ordered Dataset

Supplementary Figure 2. The black module is associated with antigen processing and presentation and T cell activation, related to Figure 3. **(A)** Plot showing the correlation matrix among the top 10 hub genes in the black module after adjusting for tumor purity. **(B)** The top 10 hub genes of the black module were downregulated in the tumor versus normal tissue by analyzing the TCGA NSCLC dataset combined with GTEx lung dataset. **(C)** Kyoto Encyclopedia of Genes and Genomes (KEGG) enrichment analysis shows the top eight enriched pathways in the black module. **(D-F)** GSEA plot against Gene Ontology (GO)/KEGG demonstrating the enrichment of **(D)** antigen processing and presentation peptide antigen (GO:0048002), **(E)** antigen processing and presentation (hsa04612), and **(F)** T cell activation (GO:0042110) in the black module. The P values in (**B**) were derived from Wilcoxon rank-sum test. ****:$P < 0.0001$. In box plots, the central line is the median, and the limits are the upper and lower quartiles.

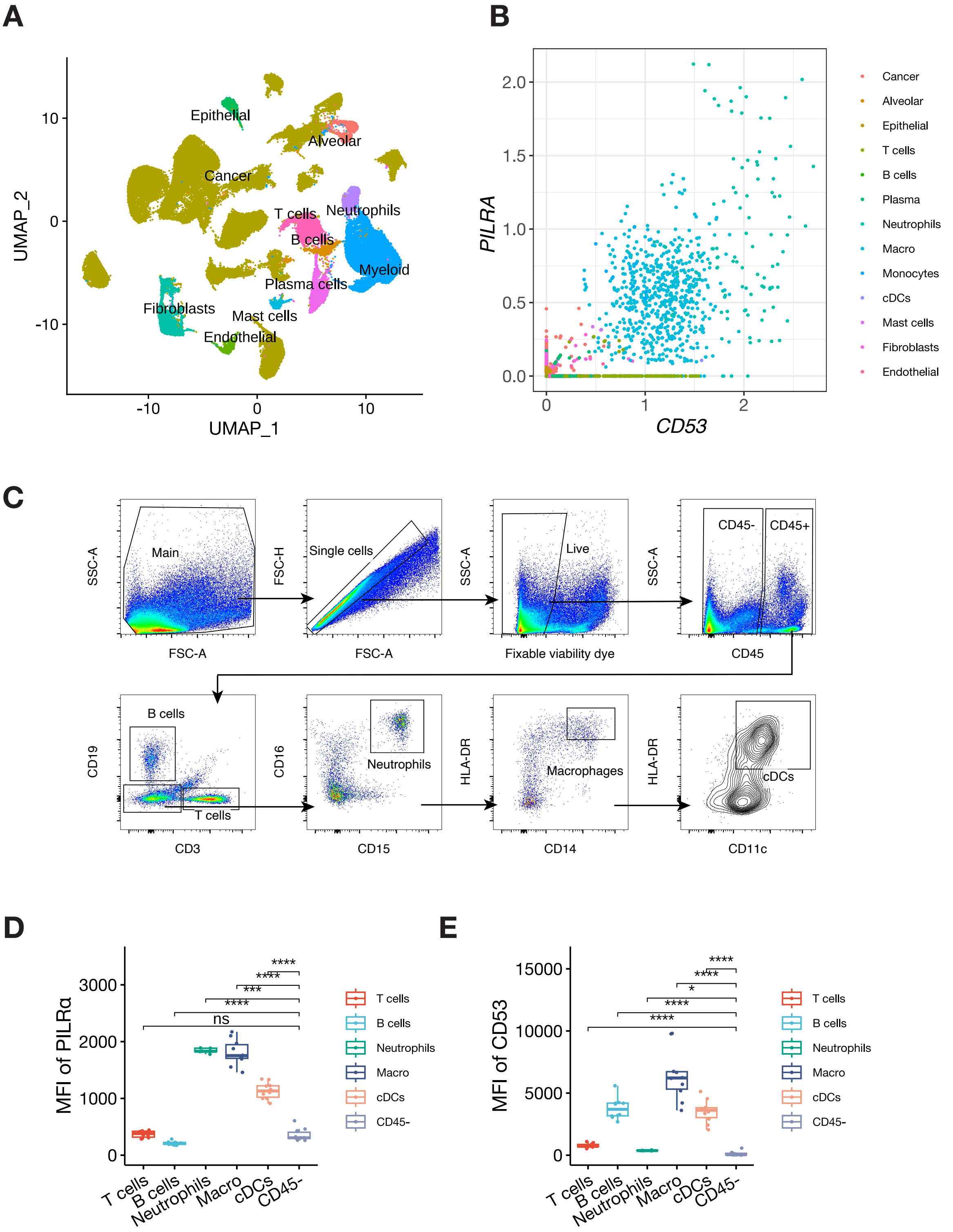

Supplementary Figure 3. The black module genes are specifically expressed in myeloid cells, related to Figure 4. **(A)** UMAP plot showing cell types based on the scRNA-seq data of NSCLC (GSE148071). **(B)** Scatter plot showing the expression of *PILRA* versus the expression of *CD53*

colored by cell types. **(C)** Gating strategy of immune cell types in NSCLC samples. **(D, E)** Box plot showing the mean fluorescent intensities (MFI) of **(D)** PILRα and **(E)** CD53 among different cell types identified by flow cytometry. The *P* values in **(D, E)** were derived from Wilcoxon rank-sum test. ns: not significant, *: $P < 0.05$, ***: $P < 0.001$, ****: $P < 0.0001$. In box plots, the central line is the median, and the limits are the upper and lower quartiles.
cDCs, conventional dendritic cells; Macro, macrophages; CD45-, $CD45^-$ non-immune cells.

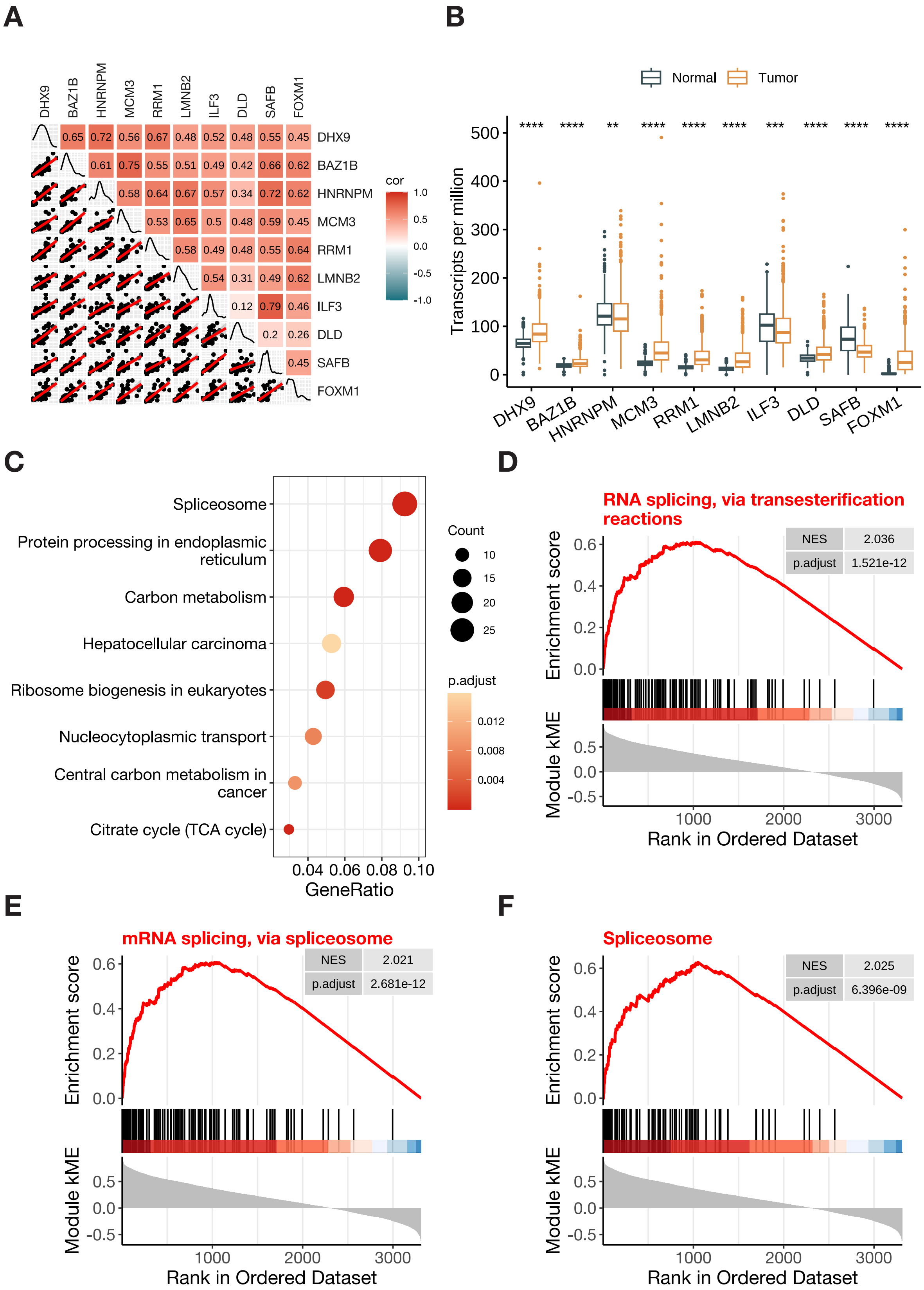

A
B
C
D
E
F
DHX9 BAZ1B HNRNPM MCM3 RRM1 LMNB2 ILF3 DLD SAFB FOXM1
cor
1.0 0.5 0.0 -0.5 -1.0
Normal Tumor
Transcripts per million
Spliceosome
Protein processing in endoplasmic reticulum
Carbon metabolism
Hepatocellular carcinoma
Ribosome biogenesis in eukaryotes
Nucleocytoplasmic transport
Central carbon metabolism in cancer
Citrate cycle (TCA cycle)
GeneRatio
Count
p.adjust
RNA splicing, via transesterification reactions
NES 2.036
p.adjust 1.521e-12
mRNA splicing, via spliceosome
NES 2.021
p.adjust 2.681e-12
Spliceosome
NES 2.025
p.adjust 6.396e-09
Enrichment score
Module kME
Rank in Ordered Dataset

Supplementary Figure 4. The blue module is associated with RNA splicing, related to Figure 5. **(A)** Plot showing the correlation matrix among the top 10 hub genes in the blue module after adjusting for tumor purity. **(B)** The top 10 hub genes of the blue module were upregulated in the tumor versus normal tissue by analyzing the TCGA NSCLC dataset combined with GTEx lung dataset. **(C)** Kyoto Encyclopedia of Genes and Genomes (KEGG) enrichment analysis shows the top eight enriched pathways in the blue module. **(D-F)** GSEA plot against Gene Ontology (GO)/KEGG demonstrating the enrichment of **(D)** RNA splicing, via transesterification reactions (GO:0000375), **(E)** mRNA splicing, via spliceosome (GO:0000398), and **(F)** spliceosome (hsa03040) in the blue module. The *P* values in (**B**) were derived from Wilcoxon rank-sum test. **:$P < 0.01$, ***:$P < 0.001$, ****:$P < 0.0001$. In box plots, the central line is the median, and the limits are the upper and lower quartiles.

A

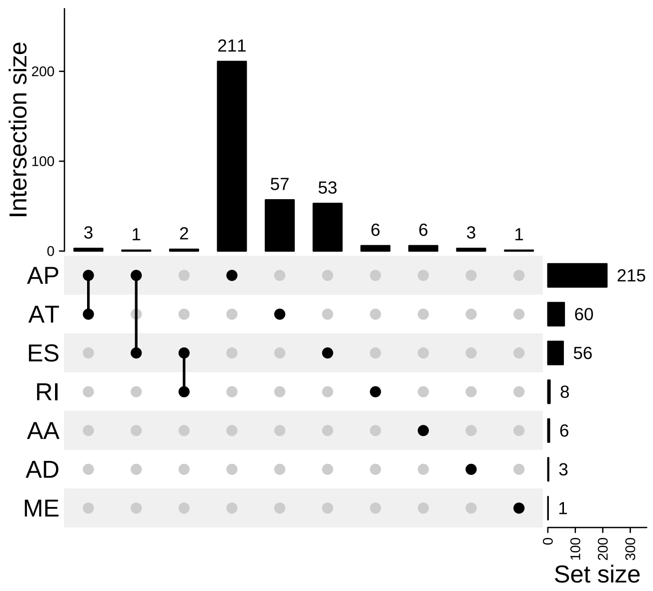

Supplementary Figure 5. Downregulated alternative splicing (AS) events in the group with high blue module scores (GSVA_blue), related to Figure 6. **(A)** UpSet plot showing the interactions between seven types of the differentially expressed AS events (DEASs) in the GSVA_blue high group.

Alternate Promoter (AP), Alternate Terminator (AT), Exon Skip (ES), Retained Intron (RI), Alternate Acceptor site (AA), Alternate Donor site (AD) and Mutually Exclusive Exons (ME).

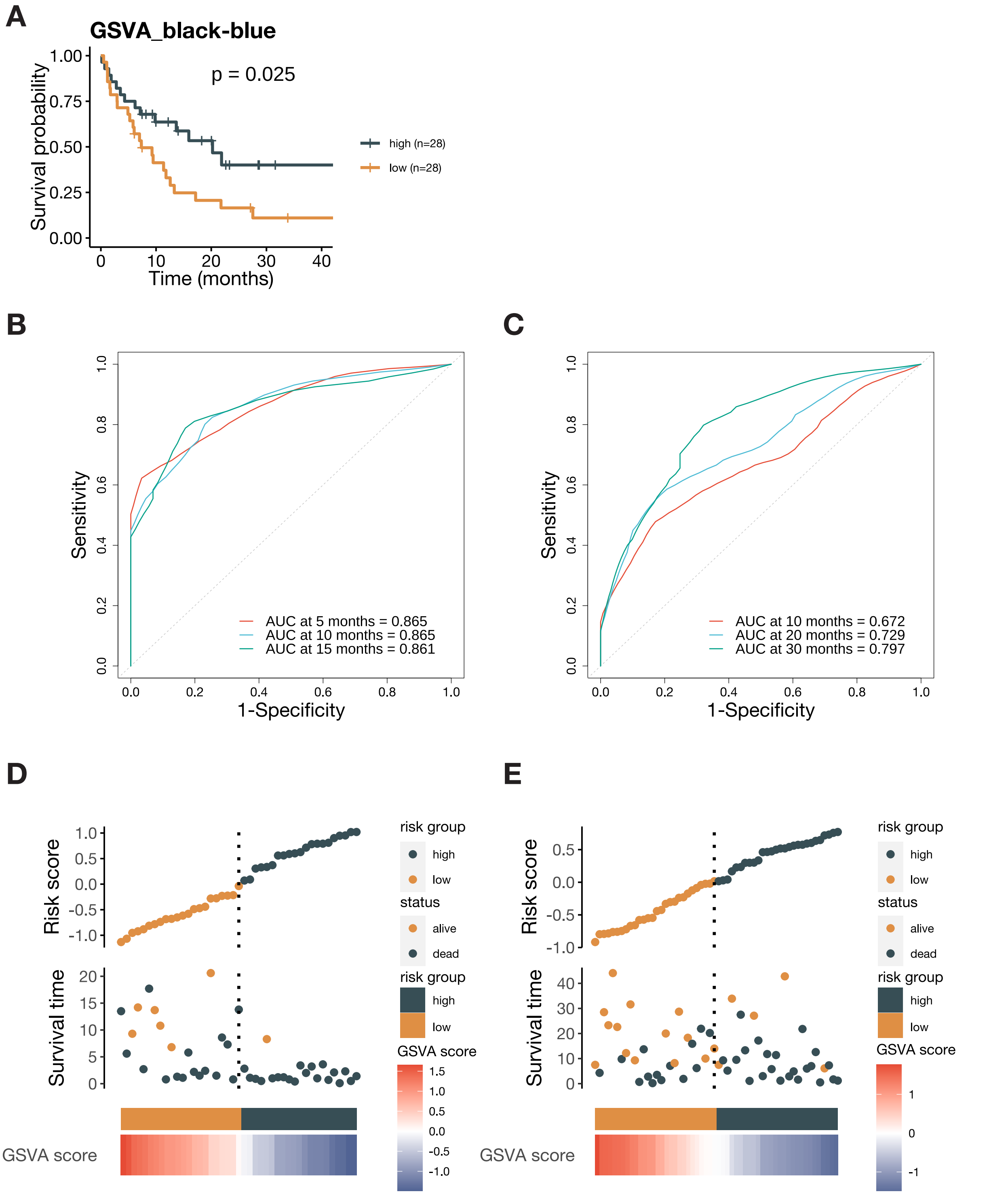

Supplementary Figure 6. GSVA score simply combines the black and blue modules, related to Figure 7. **(A)** Kaplan-Meier curve of PFS comparing patients with high GSVA_black-blue scores to those with low scores in the GLCI validation cohort (n=56). The GSVA_black-blue score was calculated by subtraction of the GSVA_blue score of each sample from the corresponding GSVA_black score. **(B)** The receiver operating characteristic (ROC) curve of PFS showing the area

under the curve (AUC) of the GSVA_black-blue score at 5, 10, 15 months in the training dataset. **(C)** The ROC curve of OS showing the AUC of the GSVA_black-blue score at 10, 20, 30 months in the GLCI validation dataset. **(D)** The distribution of the PFS and the expression of three screened genes in the GSVA_black-blue score (training dataset). **(E)** The distribution of the OS and the expression of three screened genes in the GSVA_black-blue score (GCLI validation dataset). The *P* value in **(A)** was derived from log-rank test.

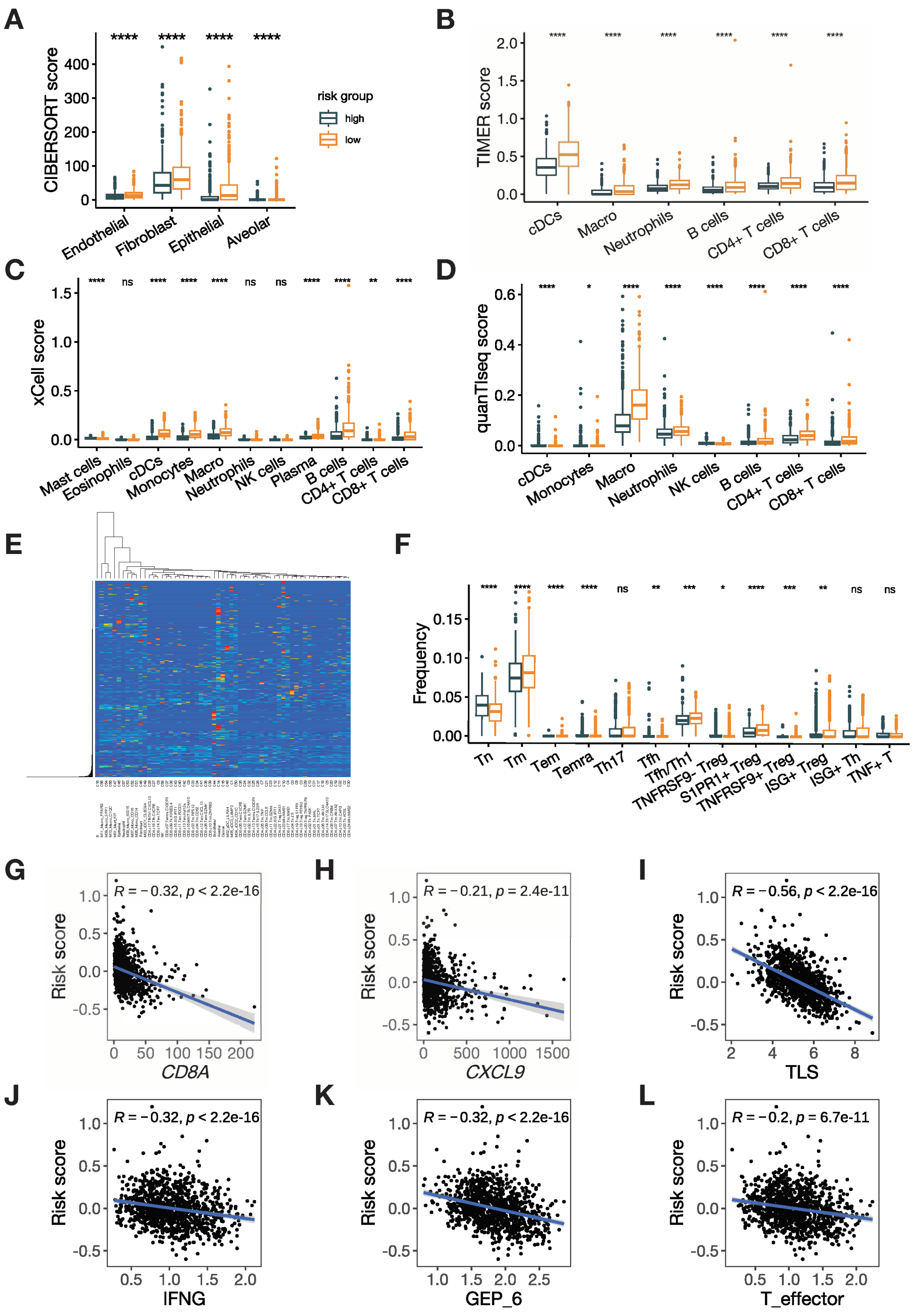
A
B
C
D
E
F
G
H
I
J
K
L
CIBERSORT score
risk group
high
low
Endothelial
Fibroblast
Epithelial
Aveolar
TIMER score
cDCs
Macro
Neutrophils
B cells
CD4+ T cells
CD8+ T cells
xCell score
Mast cells
Eosinophils
Monocytes
NK cells
Plasma
quanTIseq score
Frequency
Tn
Tm
Tem
Temra
Th17
Tfh
Tfh/Th1
TNFRSF9- Treg
S1PR1+ Treg
TNFRSF9+ Treg
ISG+ Treg
ISG+ Th
TNF+ T
Risk score
R = −0.32, p < 2.2e-16
CD8A
R = −0.21, p = 2.4e-11
CXCL9
R = −0.56, p < 2.2e-16
TLS
R = −0.32, p < 2.2e-16
IFNG
R = −0.32, p < 2.2e-16
GEP_6
R = −0.2, p = 6.7e-11
T_effector

Supplementary Figure 7. The risk score is related to immune cell composition changes and previously reported parameters predicting ICB responsiveness, related to Figure 8. **(A)** Box plot showing the cell frequencies of stromal cells using the CIBERSORTx tool run in relative mode. The signature matrix used is the customized scRNA-seq profile. (**B-D**) Box plots comparing the cell abundance of major immune cells between high- and low-risk groups using the (**B**) TIMER score, (**C**) xCell score, and (**D**) quanTIseq score. (**E**) Heatmap showing the signature matrix customized by the CIBERSORTx tool based on the Bernard_Thienpont NSCLC scRNA-seq. **(F)** Box plot showing the cell frequencies of $CD4^+$ T cells between high- and low-risk groups using the CIBERSORTx tool run in relative mode. The signature matrix used is the customized scRNA-seq profile. **(G-L)** Scatter plots illustrating the correlations of **(G)** *CD8A* expression, **(H)** *CXCL9* expression, **(I)** tertiary lymphoid structure (TLS) signature, **(J)** IFNG signature, **(K)** GEP_6 signature and **(K)** T_effector signature versus the risk score. Data in **(A-D, F-L)** were analyzed using the TCGA NSCLC dataset. The *P* values in **(B, C)** were derived from Wilcoxon rank-sum test. ns: not significant, *: $P < 0.05$, **: $P < 0.01$, ***: $P < 0.001$, ****: $P < 0.0001$. In box plots, the central line is the median, and the limits are the upper and lower quartiles.

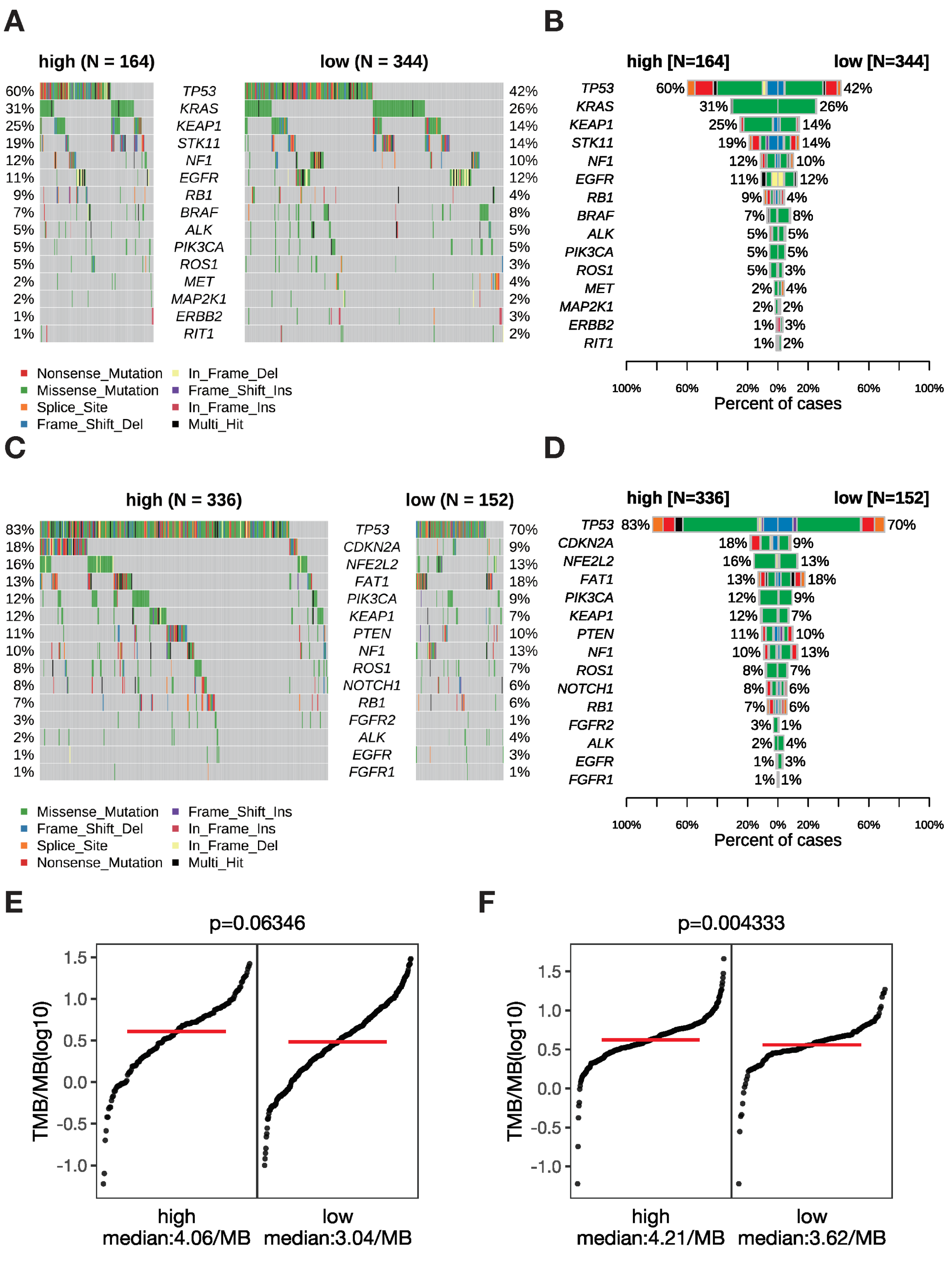

Supplementary Figure 8. Mutational changes between high- and low-risk groups, related to Figure 8. **(A)** Co-mutation plots and **(B)** bar plots comparing the common somatic mutations in lung adenocarcinoma (LUAD) between high- and low-risk groups. **(C)** Co-mutation plots and **(D)** bar plots comparing the common somatic mutations in lung squamous cell carcinoma (LUSC) between

high- and low-risk groups. The TMB changes between high- and low-risk groups of **(E)** LUAD and **(F)** LUSC. All data in **(A-F)** were analyzed using the TCGA NSCLC dataset. The *P* values in **(E, F)** were derived from Wilcoxon rank-sum test.
